# Global trends and emerging frontiers in neutrophil extracellular trap-related sepsis research: a bibliometric analysis

**DOI:** 10.3389/fimmu.2026.1954033

**Published:** 2026-10-26

**Authors:** Tiantian Xu, Guangbin Yu, Minqi Lu, Qinlong Gao, Qi Chen, Linyan Liang, Jingjiao Hong, Wenjie Xu, Dan Xi

**Affiliations:** 1 Shenzhen Bao’an Chinese Medicine Hospital, Guangzhou University of Chinese Medicine, Shenzhen, China; 2 The Second Clinical College of Guangzhou University of Chinese Medicine, Guangzhou University of Chinese Medicine, Guangdong, China; 3 Clinical Medical School of Acupuncture, Moxibustion and Rehabilitation, Guangzhou University of Chinese Medicine, Guangdong, China; 4 The First Affiliated Hospital of Guangzhou University of Chinese Medicine, Guangdong, China

**Keywords:** bibliometric analysis, immunothrombosis, knowledge mapping, neutrophil extracellular traps, sepsis

## Abstract

**Background:**

Sepsis remains a major challenge in critical care medicine, and NETs have emerged as important regulators linking inflammation, immunity, coagulation, and tissue injury. However, the global development trajectory and research frontiers of NET-related sepsis studies remain unclear.

**Methods:**

Publications related to NET-related sepsis research from 2004 to September 19, 2026, were independently retrieved from the Web of Science Core Collection and Scopus databases. After database-specific screening, 263 WoSCC records and 499 Scopus records were retained for separate analyses using VOSviewer and CiteSpace to evaluate publication trends, collaboration networks, knowledge structures, thematic evolution, and emerging research attention.

**Results:**

The WoSCC and Scopus analytical datasets contained 263 and 499 records, respectively. WoSCC analysis showed that China had the highest publication output, whereas the United States had the highest citation impact and collaboration strength among the listed countries. Cleaned author-keyword analysis highlighted sepsis, neutrophil extracellular traps, NETosis, inflammation, neutrophil, platelet, acute lung injury, and septic shock. Cross-database comparison showed a high correlation in complete-year publication trends (Spearman’s ρ = 0.986) and partial overlap in high-frequency author keywords.

**Conclusion:**

Independent database analyses indicate sustained growth and a progressively interconnected knowledge structure in NET-related sepsis research. The results describe publication, collaboration, citation, and thematic attention patterns; they do not establish biomarker performance or therapeutic efficacy. Future studies may benefit from standardized NET measurement and reporting, multicenter validation of NET-related biomarkers and predictive models, and appropriately designed clinical investigations.

## 1 Introduction

Sepsis is a life-threatening syndrome caused by a dysregulated host response to infection and remains a major challenge in critical care medicine. The Third International Consensus Definitions for Sepsis and Septic Shock (Sepsis-3) emphasized that organ dysfunction results not only from the invading pathogen but also from maladaptive host responses to infection (1). These responses involve interconnected changes in innate and adaptive immunity, endothelial function, coagulation, metabolism, and tissue homeostasis (2, 3). The biological and clinical heterogeneity of sepsis has limited the effectiveness of uniform diagnostic and therapeutic approaches, highlighting the need to identify mechanisms and biomarkers that can better characterize distinct host-response states. Neutrophils are major components of the early innate immune response and contribute to pathogen control through phagocytosis, degranulation, and reactive oxygen species production. The discovery of NETs expanded this conventional view of neutrophil function. Brinkmann et al. demonstrated that activated neutrophils release extracellular chromatin structures decorated with histones and antimicrobial proteins, which can capture and restrict microorganisms (4). Subsequent studies showed that NET formation is highly context dependent. Although NETs may support antimicrobial defense, excessive formation, impaired degradation, or prolonged intravascular persistence can amplify inflammation, injure endothelial and parenchymal cells, and contribute to microvascular dysfunction (5, 6).

The association between NETs and immunothrombosis has further increased interest in their role in sepsis. Immunothrombosis describes the coordinated interaction between innate immunity and coagulation that helps contain invading pathogens but may become harmful when excessively or persistently activated (7). NETs can provide a scaffold for interactions among neutrophils, platelets, coagulation factors, complement components, and endothelial cells, thereby linking inflammatory activation with thrombotic and microcirculatory alterations (8–10). Studies have consequently investigated NETs in relation to organ injury, disease severity, prognosis, biomarker development, and potential therapeutic targets (11). Nevertheless, the protective and injurious effects of NETs may vary according to pathogen type, disease stage, host immune status, and NET clearance capacity, complicating their clinical interpretation.

The literature on NETs and sepsis has expanded rapidly and now spans immunology, hematology, vascular biology, infectious diseases, and critical care medicine. Narrative and systematic reviews have summarized specific molecular mechanisms or clinical implications of NETs; however, these approaches are not designed to quantify the global development of the field, identify influential contributors and intellectual foundations, or trace the temporal migration of research themes. Bibliometric analysis complements conventional evidence synthesis by examining publication growth, citation relationships, collaboration networks, co-cited knowledge structures, keyword evolution, and emerging topics (12). Previous bibliometric studies have examined NETs across broad disease categories or evaluated general trends in sepsis and inflammatory research (13–16). However, the global structure and temporal evolution of NET-related sepsis research have not been comprehensively characterized using an integrated analysis of publication output, international collaboration, intellectual foundations, and emerging clinically oriented themes. A disease-specific bibliometric assessment may therefore help clarify how foundational studies of neutrophil biology have developed into research on immunothrombosis, organ dysfunction, biomarkers, risk prediction, and therapeutic modulation.

Accordingly, this study analyzed publications retrieved from the Web of Science Core Collection (WoSCC) and Scopus databases from January 1, 2004 to September 19, 2026, using VOSviewer (v1.6.20) and CiteSpace (v6.4.R1). The objectives were to (1): characterize temporal publication trends and the contributions of countries, institutions, journals, and authors (2); map international and institutional collaboration patterns (3); identify the major intellectual foundations, knowledge clusters, and thematic evolution of the field; and (4) determine emerging research frontiers and unresolved translational questions in NET-related sepsis research.

## 2 Materials and methods

### 2.1 Data sources and search strategy

The data for this study were retrieved independently from two major academic databases: the Web of Science Core Collection (WoSCC, https://www.webofscience.com/wos/woscc/basic-search) and Scopus (https://www.scopus.com). Considering that WoSCC and Scopus have different indexing systems, journal coverage, and citation calculation mechanisms, the two databases were analyzed separately rather than integrated into a single bibliographic dataset. This database-specific analytical strategy was adopted to improve methodological transparency and reproducibility and to minimize potential bias caused by differences in database coverage and citation metrics.

The retrieval period for both databases was restricted from January 1, 2004, to September 19, 2026. The starting year was selected based on the initial description of neutrophil extracellular traps (NETs), ensuring that the retrieved publications corresponded to the established NET terminology. All literature searches and data export procedures were completed on September 19, 2026, to minimize potential bias caused by continuous database updates. Records retrieved from each database were exported separately for subsequent screening and database-specific bibliometric analyses.

#### 2.1.1 Search strategy for WoSCC

The search strategy for WoSCC was as follows:

TS = ((“Neutrophil Extracellular Trap” OR “neutrophil extracellular DNA traps” OR “NETosis”) AND (“Sepsis” OR “Septic Shock” OR “septicaemia” OR “Septicemia”)).

The search strategy retrieved 495 records from WoSCC. After restricting document types to articles and review articles, 469 records remained. All retained records were published in English; therefore, no additional exclusion based on language was required. Two independent reviewers subsequently screened the 469 records by evaluating titles, abstracts, and full texts according to predefined inclusion and exclusion criteria. Studies were excluded if they met any of the following criteria (1): non-sepsis-related studies (2); studies without relevance to NETs (3); studies with only background information or limited relevance to the research topic; or (4) inappropriate publication types. Any disagreement between reviewers was resolved through discussion and consensus. A total of 263 publications were included in the WoSCC-based bibliometric analysis, comprising 185 articles and 78 reviews (**Figure 1**).

**Figure 1:**
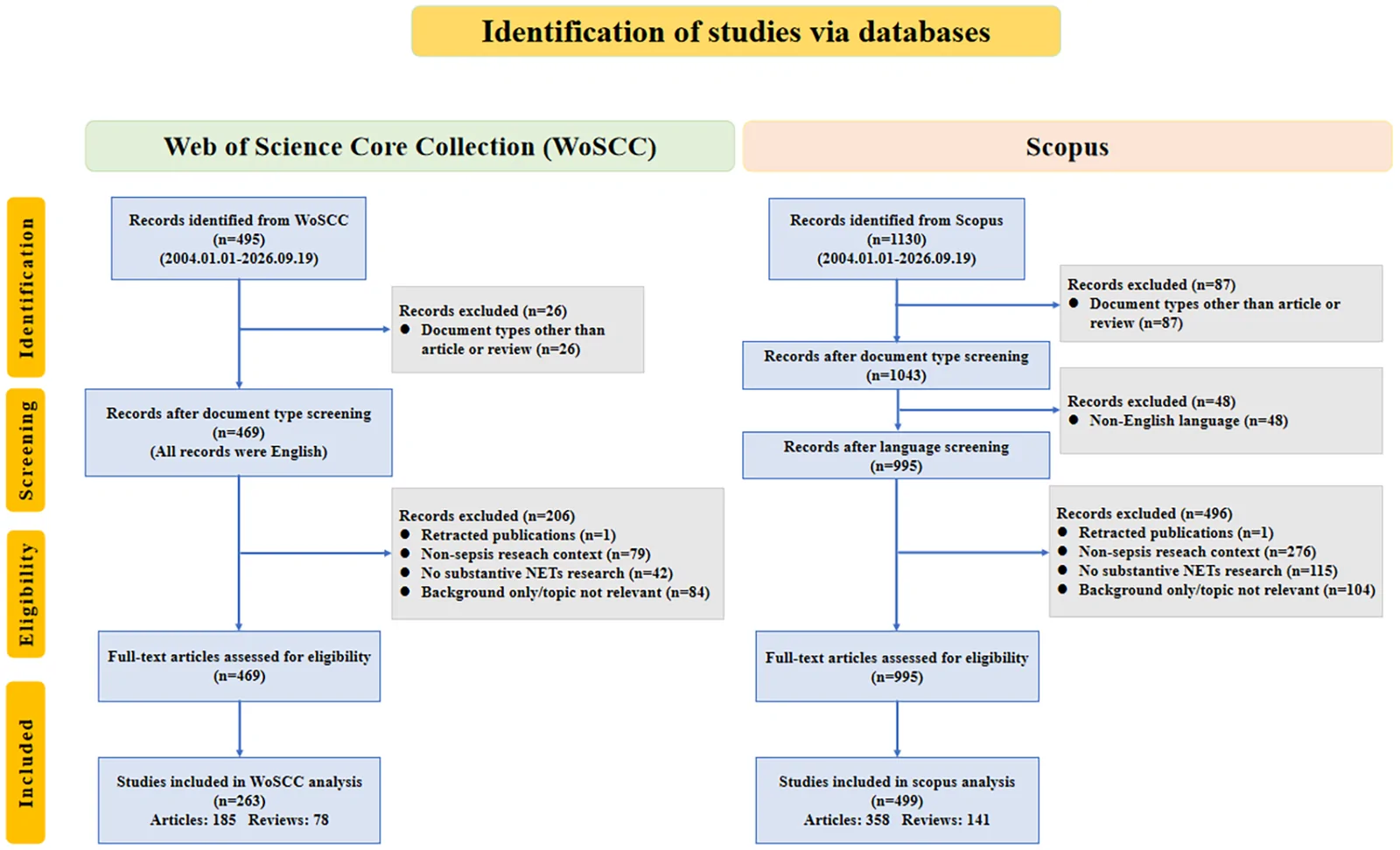
PRISMA 2020 flow diagram of study identification, screening, eligibility assessment, and inclusion. The flow diagram presents the independent screening processes for WoSCC (left) and Scopus (right), including records identified, excluded after document-type screening, excluded after language screening (Scopus only), excluded after full-text assessment, and finally included in each database-specific analysis. WoSCC, Web of Science Core Collection.

#### 2.1.2 Search strategy for Scopus

The search strategy for Scopus was as follows:

TITLE-ABS-KEY ((“Neutrophil Extracellular Trap” OR “neutrophil extracellular DNA traps” OR “NETosis”) AND (“Sepsis” OR “Septic Shock” OR “septicaemia” OR “Septicemia”)).

A total of 1,130 records were initially retrieved from Scopus. After restricting document types to articles and reviews, 1,043 records remained. After applying the language criterion, 995 English-language records were retained for further screening. Two independent reviewers screened the 995 records by assessing titles, abstracts, and full texts according to the predefined eligibility criteria. Studies were excluded if they were unrelated to sepsis, did not investigate NETs, contained only background or peripheral information, or did not meet the predefined publication criteria. Disagreements were resolved through discussion and consensus. Finally, 499 publications were retained for the Scopus-based bibliometric analysis, consisting of 358 articles and 141 reviews (**Figure 1**).

#### 2.1.3 Study selection and data processing

The screening and selection processes for WoSCC and Scopus were conducted independently. Duplicate removal was not performed to create a merged analytical dataset because the two databases represent independent citation ecosystems. Instead, each database-derived dataset was separately processed and analyzed. The detailed study identification, screening, eligibility assessment, and inclusion procedures for both databases are presented in the revised PRISMA 2020 flow diagram (**Figure 1**). All included records were exported in plain text format for WoSCC and CSV format for Scopus to ensure data consistency and reproducibility.

#### 2.1.4 Keyword preprocessing

For keyword analyses, only author keywords were used: the Author Keywords (DE) field in WoSCC and the Author Keywords field in Scopus. Keywords Plus (ID) (WoSCC) and Index Keywords (Scopus) were excluded because they are database-generated indexing terms that can introduce generic descriptors such as “humans,” “article,” and “controlled study,” which do not represent scientific themes. Separate thesaurus files were constructed for the WoSCC and Scopus datasets to harmonize singular/plural forms, spelling variants, and synonymous expressions. The detailed keyword preprocessing rules are provided in **Supplementary Tables S1**, **S2**. Generic indexing terms, document-type descriptors, and overly broad terms that could not distinguish specific research directions were removed by leaving the “replace by” column empty in the thesaurus file. In CiteSpace, only the DE term source was selected, and ID was deselected. The same thesaurus rules were applied to the Scopus dataset. The cleaned DE lists were then used for co-occurrence, clustering, timeline, and burst analyses.

### 2.2 Mapping tools and bibliometric indicators

This study employed VOSviewer and CiteSpace to perform visualization and analysis of the 263 WoSCC documents. VOSviewer is a software tool specifically designed for constructing and visualizing knowledge maps from bibliometric network data (17). Through systematic analysis of citation data, this tool constructs co-citation networks, collaboration networks, and citation networks, providing intuitive and clear visual representations of the knowledge structure within a specific research field. In this study, VOSviewer was primarily used to map and visualize association networks at the country, institution, journal, and author levels (18).

CiteSpace is a Java-based scientific knowledge mapping tool with broad influence in the field of information visualization. It remains one of the most representative and highly cited tools in this domain and is widely applied in the mining of academic patterns and the prediction of research frontiers (19). Accordingly, this study used VOSviewer to construct bibliographic coupling network maps among authors, journals, and countries, and employed CiteSpace to visualize the knowledge base and research hotspots in the field of NET-related sepsis, as well as to explore potential emerging topics through burst detection.

In bibliometric analysis, the number of publications (NP) and the number of citations (NC) are commonly used indicators. NP quantifies research output, whereas NC reflects citation impact (20). The H-index combines publication output and citation impact: a researcher has an H-index of H if at least H papers have each received at least H citations. Although originally designed for individual researchers, it has been extended to compare countries/regions, institutions, and journals as a bibliometric impact indicator, not as a direct measure of research quality (21). In this study, H-index values for countries/regions, institutions, journals, and authors were derived exclusively from the WoSCC dataset. For each entity, a corresponding WoSCC record set was created by refining the search results using the relevant field (country/region, organization-enhanced, source title, or author). A citation report was then generated in WoSCC, and the H-index was read directly from the report. The citation counts used for H-index calculation were based on WoSCC data as of September 19, 2026. H-index values were not calculated from Scopus, and no citation metrics were merged across the two databases. In this study, H-index is reported as a bibliometric impact indicator that combines publication output and citation impact, and it is not interpreted as a direct measure of research quality.

The Journal Impact Factor (JIF), based on the latest Journal Citation Reports (JCR), is a journal-level citation impact indicator (22). It does not measure the quality of individual articles and was therefore not used to infer article-level quality. In this study, “unique first authors” refers to the deduplicated number of first authors among the included publications. No minimum publication threshold was applied; the count was restricted to first authors only.

VOSviewer was used to construct and visualize bibliometric network maps, presenting associative relationships among academic entities such as authors, journals, and keywords (18, 23). In co-citation and co-occurrence analyses, node size corresponds to publication volume, link thickness reflects relationship strength, and node color distinguishes different clusters or time periods. CiteSpace employs multiple methods, including cluster analysis, timeline/timezone views, dual-map overlay, and citation burst detection, to generate visual maps of knowledge domains and their evolutionary trajectories, thereby revealing core research themes and potential emerging topic (24). Cluster analysis identifies fundamental research themes and emerging research trends in NET-related sepsis research through systematic categorization of high-frequency author keywords and references.

### 2.3 Key parameter settings of bibliometric tools

This study employed CiteSpace and VOSviewer to perform bibliometric analysis and knowledge map visualization.

Regarding the parameter settings of CiteSpace: the time span was set from 2013 to September, 19 2026, with a time slice unit of 1 year based on the temporal coverage of the retained WoSCC records; node selection adopted the g-index method (k = 5); the remaining parameters were configured as follows: Link Retaining Factor (LRF) = 2.5, maximum number of links per node (L/N) = 10, Look-Back Years (LBY) = 5, and the topological pruning coefficient (E) = 1.0. Cluster analysis was performed using the Log-Likelihood Ratio (LLR) algorithm. Network size is reported in terms of the number of nodes (N) and edges (E), and clustering quality is evaluated using the modularity value Q and the weighted mean silhouette value S; a clustering structure is considered significant and robust when Q > 0.3 and S > 0.7.

Concerning the parameter settings of VOSviewer: network visualization analyses were sequentially conducted for countries, institutions, journals, and co-cited references, with appropriate minimum occurrence thresholds (e.g., minimum number of documents for countries/institutions = 5; minimum occurrences for author keywords = 10) and attraction/repulsion parameters set for each analysis type based on the actual distribution characteristics of the data. Data normalization adopted the fractional counting method, entity similarity was calculated using the cosine metric, and clustering employed the software’s built-in density-based default clustering algorithm (18). In the network visualization presentation, node size is proportional to term frequency/publication volume, and label size is proportional to total link strength; the spatial layout is generated by the VOS core layout algorithm to ensure map readability and clarity. For keyword co-occurrence analysis, the cleaned author-keyword dataset was used, and the thesaurus file was loaded to ensure consistent terminology.

### 2.4 Cross-database concordance assessment

To evaluate whether the main WoSCC-based findings were robust across databases, the independently screened Scopus dataset was analyzed separately and compared with the WoSCC results at the pattern level. No records or citation metrics were merged across the two databases.

The following concordance dimensions were assessed:

1. Annual publication trend: annual publication counts from WoSCC and Scopus were compared for the common period 2013-2026. Spearman’s rank correlation coefficient (ρ) was calculated based on complete years 2013–2025 to quantify the consistency of temporal patterns. The 2026 data were incomplete (search completed on September 19, 2026) and were therefore excluded from the concordance analysis.
2. High-frequency author keywords: the top 20 author keywords from each database were compared. Overlap was visualized using a Venn diagram, and the Jaccard similarity coefficient was calculated as the number of shared keywords divided by the number of unique keywords in the union.

In addition, Scopus-based keyword co-occurrence, keyword clustering, country collaboration, and institution collaboration networks were generated independently using VOSviewer and CiteSpace and are provided as **Supplementary Figures S1**-**S4**. These analyses provided an independent cross-database comparison of thematic and collaboration patterns.

### 2.5 Ethical considerations

Because this study analyzed publicly available bibliographic records and did not involve human participants, experimental animals, or identifiable personal information, ethical approval was not required.

## 3 Results

### 3.1 Global publication trends and database-specific growth

The screened analytical datasets comprised 263 WoSCC records (185 articles and 78 reviews) and 499 Scopus records (358 articles and 141 reviews). These counts refer to records retained after database-specific screening and were analyzed separately; no records or citation metrics were merged.

Annual publication counts from both databases showed an overall increasing trend from 2013 to 2025 (**Figure 2**). The Spearman rank correlation coefficient between WoSCC and Scopus annual publication counts was ρ = 0.986 (based on complete years 2013-2025), indicating a high degree of concordance in temporal trends. The 2026 data were incomplete (search completed on September 19, 2026) and were therefore not included in the concordance analysis.

**Figure 2:**
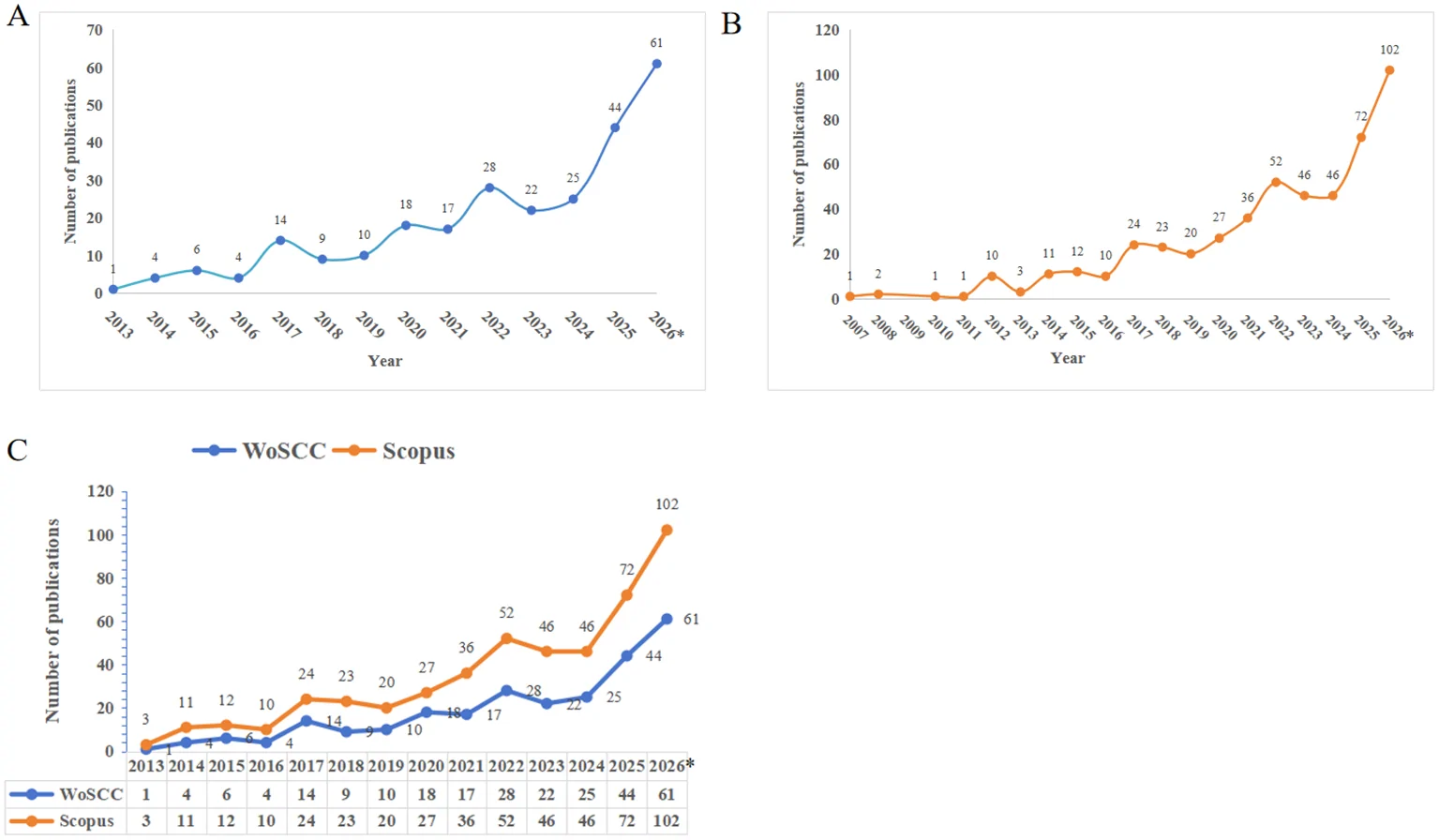
Annual publication trends. **(A)** Annual publication counts from WoSCC (2013-2026). **(B)** Annual publication counts from Scopus (2007-2026). **(C)** Comparative annual publication trends from WoSCC (blue) and Scopus (orange) for the common period 2013-2026. The earliest included Scopus record was from 2007, whereas the earliest included WoSCC record was from 2013; early Scopus records from 2007–2012 were not included in the cross-database concordance analysis. The 2026 data are incomplete (search completed on September 19, 2026) and were not directly compared with complete years. Spearman’s rank correlation coefficient based on complete years 2013–2025 was ρ = 0.986, indicating a high degree of concordance in temporal trends. WoSCC, Web of Science Core Collection.

In the WoSCC dataset, the earliest included record was from 2013, and annual output remained modest before 2019 (**Figure 2A**). From 2013 to 2019, annual output fluctuated between 1 and 14 papers. A steady increase began in 2020 (18 papers), followed by a notable rise in 2022 (28 papers) and 2025 (44 papers). By September 19, 2026, 61 publications had been retrieved; because 2026 represented an incomplete year, this value was not interpreted as an annual publication count. In the Scopus dataset (**Figure 2B**), early records appeared from 2007 onward, with a gradual increase from 2013 (3 papers) to 2019 (20 papers), followed by a marked expansion after 2020 (27 papers), reaching 52 papers in 2022 and 72 papers in 2025. In 2026, 102 papers were published by September 19. Detailed annual publication counts for these early Scopus records (2007–2012) are provided in **Supplementary Table S3**.

### 3.2 Country contributions and collaboration networks

Country-level indicators were calculated from the WoSCC corpus and are reported as publication output (NP), citation impact (NC and H-index), and international collaboration strength (total link strength, TLS; **Table 1**). China had the highest publication output (NP = 130), followed by the United States (NP = 69). The United States had the highest listed NC (3,812), H-index (43), and TLS (47), whereas China had NC = 2,376, H-index = 34, and TLS = 31.

**Table 1:** Top 10 countries/regions with the highest number of publications.

Rank	Country	NP	NC	Total Link Strength	H-index
1	China	130	2376	31	34
2	USA	69	3812	47	43
3	Japan	16	511	16	17
4	Germany	12	391	6	17
5	United Kingdom	11	738	8	18
6	Canada	10	949	8	14
7	South Korea	10	465	5	12
8	France	8	424	3	12
9	Spain	8	55	7	9
10	Poland	6	168	1	7

Japan, Germany, the United Kingdom, Canada, South Korea, France, Spain, and Poland also appeared among the ten countries with the highest publication output. The ranking therefore separated publication volume from citation impact and collaboration strength without treating any of these indicators as a direct measure of research quality.

The country collaboration network (**Figure 3A**) placed the United States at the most connected position, with China forming another major node. European countries showed dense regional connections, and the map included a prominent United States-China collaboration link. The geographic distribution map (**Figure 3B**) showed that the analyzed output was concentrated in North America, Europe, and East Asia. Country NP values were based on full counting, so a publication with authors from multiple countries could contribute to more than one country total.

**Figure 3:**
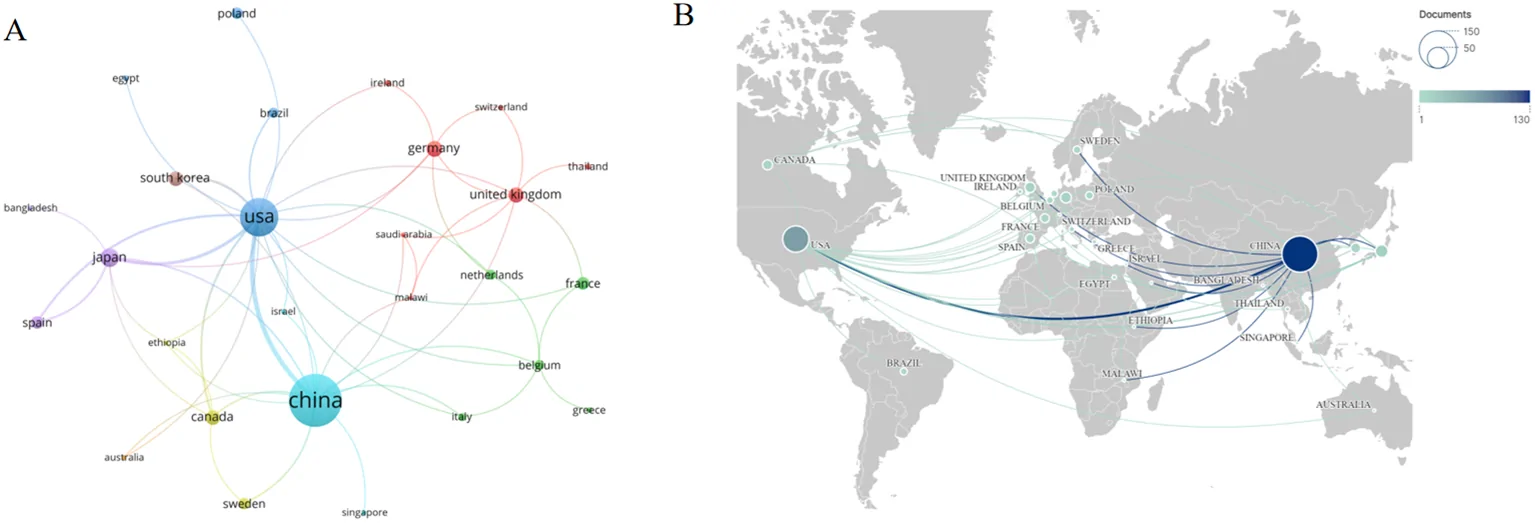
Geographic distribution and international collaboration of NET-related sepsis research. **(A)** International collaboration network. **(B)** Global map of publication outputs and collaboration links. Publications are unevenly distributed geographically, with clear regional concentrations in North America, Europe, and East Asia. Node size in **(A)** corresponds to publication volume, and line thickness denotes collaboration intensity. NET, neutrophil extracellular trap.

Together, these results describe a geographically concentrated field with distinct patterns of publication output, citation impact, and international collaboration.

### 3.3 Institutional publication and collaboration patterns

Institution-level analyses were performed on the WoSCC corpus using NP, NC, TLS, and H-index (**Table 2**). Chinese institutions occupied eight of the ten listed positions. Central South University had the highest NP (13) and TLS (19), while Fudan University ranked second in NP (10). The University of Michigan had the highest NC (470) and H-index (13) among the listed institutions. Harvard Medical School had NP = 8, NC = 231, TLS = 19, and H-index = 10. The remaining listed institutions had NP values from 6 to 7, with citation and collaboration indicators varying across institutions.

**Table 2:** Top 10 institutions with the highest number of publications.

Rank	Institution	Country	NP	NC	Total Link Strength	H-index
1	Central South University	China	13	213	19	10
2	Fudan University	China	10	221	15	8
3	Harvard Medical School	USA	8	231	19	10
4	Nanjing Medical university	China	7	60	7	5
5	University of Michigan	USA	7	470	16	13
6	Capital Medical University	China	6	19	9	4
7	Nanjing University	China	6	178	7	6
8	Naval Medical University	China	6	153	5	6
9	Shanghai Jiao Tong University	China	6	374	4	5
10	Shanghai Key Lab Perioperat Stress & Protect	China	6	156	11	5

The institutional collaboration map (**Figure 4**) showed several connected groups centered on Chinese universities, University of Michigan and Harvard Medical School, and other international institutions. Node size represented publication output, whereas link density represented collaboration strength. Central South University and Harvard Medical School both had TLS = 19, while Fudan University had TLS = 15. The network therefore contained both domestic institutional clusters and cross-regional connections.

**Figure 4:**
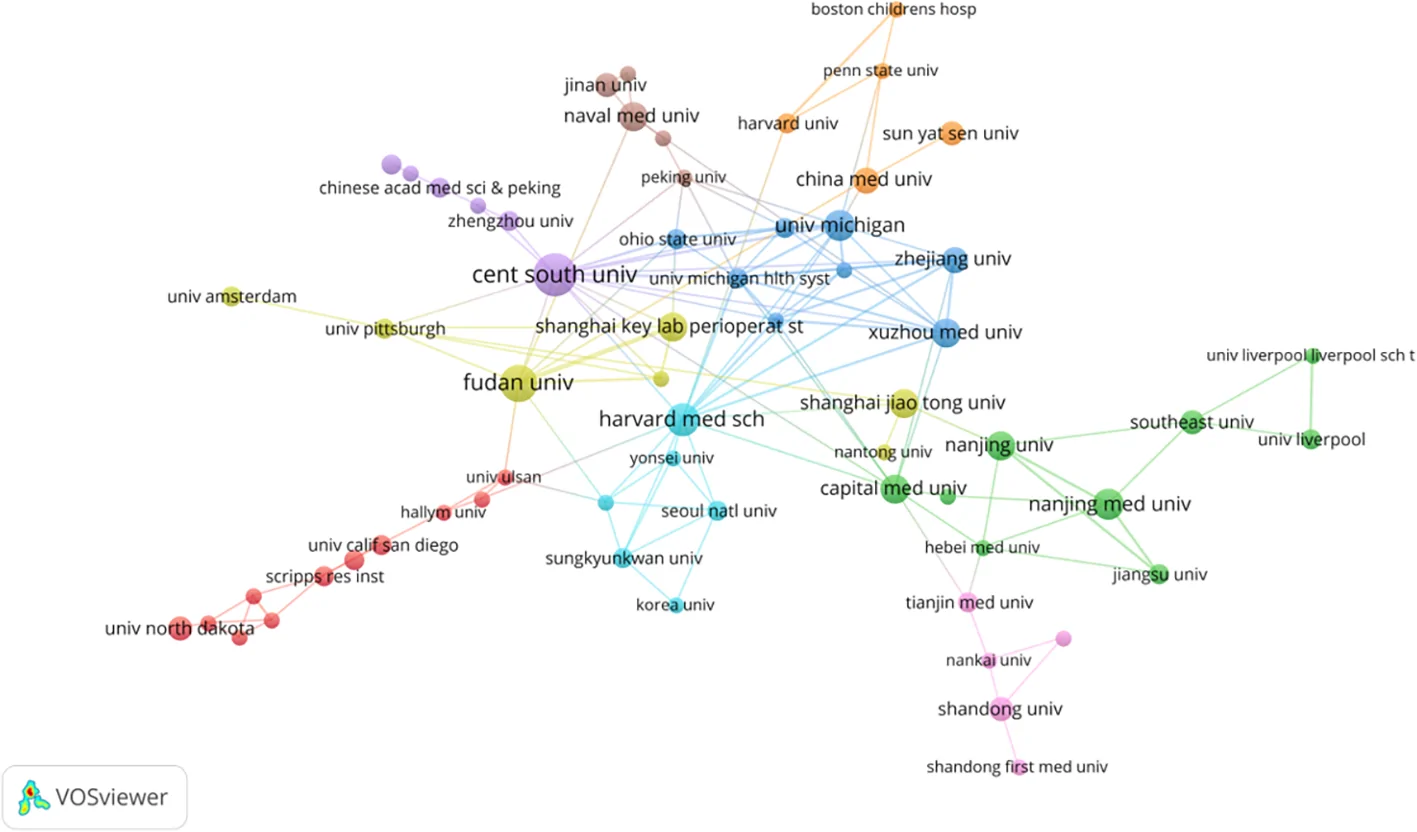
Collaboration network of institutions. Node size corresponds to publication volume, and line thickness denotes relationship intensity. Node colors distinguish different clusters of collaboration. The network exhibits a pronounced core–periphery structure.

These patterns indicate concentration of publication output in a limited set of institutions, together with heterogeneous citation impact and collaboration strength.

### 3.4 Journal publication and citation analysis

Journal-level analyses used NP, NC, TLS, H-index, and the 2025 JIF reported in **Table 3**. *Frontiers in Immunology* was the most productive listed journal (NP = 26) and also had the highest listed NC (470), TLS (103), and H-index (21). It was followed by *International Immunopharmacology* (NP = 13) and *International Journal of Molecular Sciences* (NP = 11). Nine of the ten listed journals were classified as JCR Q1, and one was classified as Q2.

**Table 3:** Top 10 journals with the highest number of publications.

Rank	Journal	2025 IF	JCR	NP	NC	Total Link Strength	H-index
1	Frontiers in Immunology	7.0	Q1	26	470	103	21
2	International Immunopharmacology	5.6	Q1	13	154	36	8
3	International Journal of Molecular Sciences	4.9	Q1	11	328	65	14
4	Journal of Thrombosis and Haemostasis	5.0	Q1	7	291	29	6
5	Advanced Science	14.1	Q1	6	112	13	5
6	Scientific Reports	3.9	Q1	6	201	21	9
7	Shock	2.7	Q1	6	313	49	9
8	Jci Insight	6.8	Q1	5	212	20	6
9	Blood	23.9	Q1	4	276	29	4
10	Inflammation	5.4	Q2	4	71	23	3

The journal co-occurrence network (**Figure 5A**) connected immunology, molecular biology, hematology, and critical care journals. The co-citation map (**Figure 5B**) and dual-map overlay (**Figure 5C**) showed citation links from molecular and biomedical research journals toward clinical and health-related domains, indicating interdisciplinary knowledge flow.

**Figure 5:**
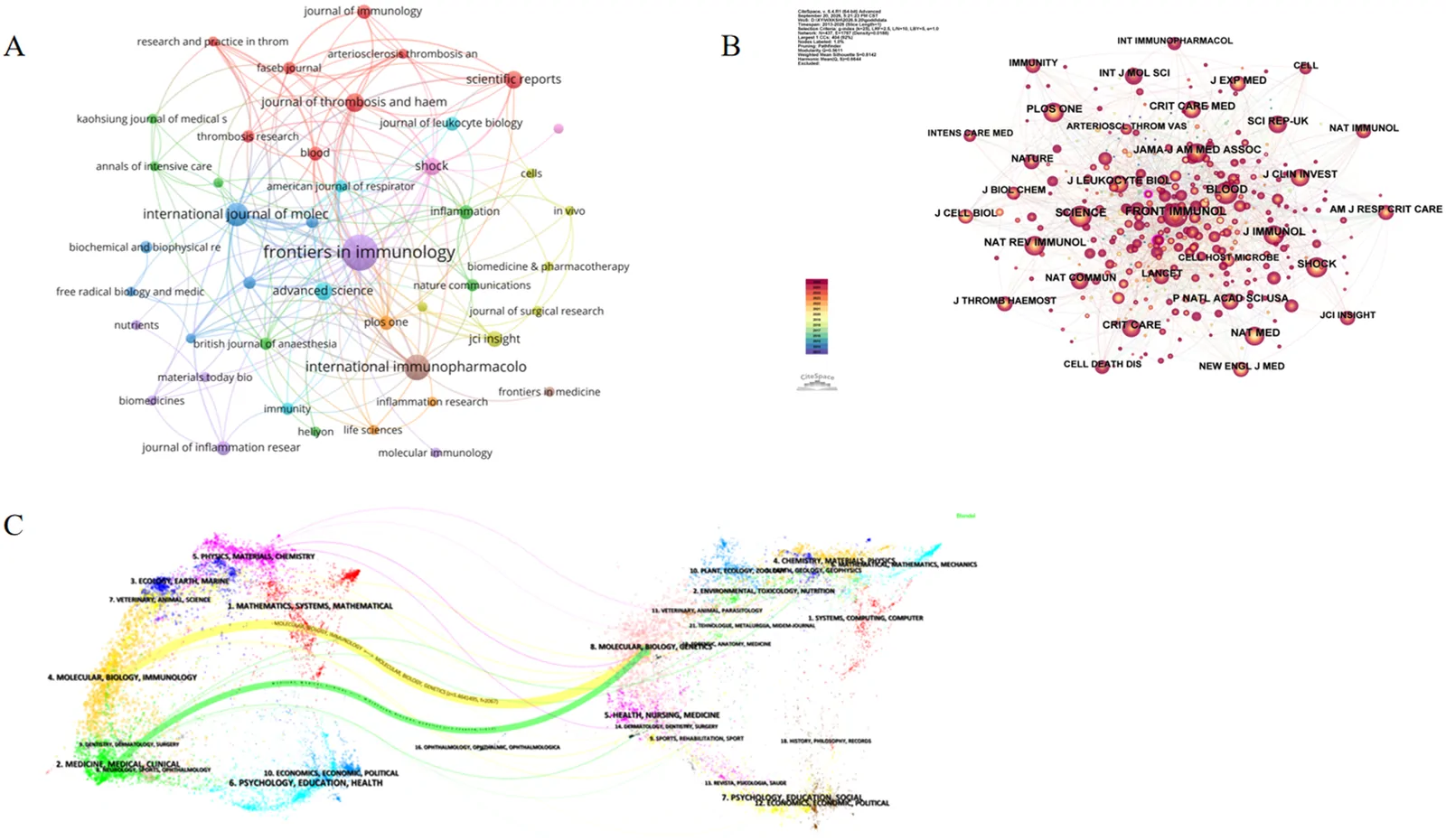
Journal impact and disciplinary knowledge flow. **(A)** Journal co-occurrence network. **(B)** Journal co-citation network. **(C)** Dual-map overlay. The colored lines represent citation connections, with the left side indicating the citing domains and the right side showing the cited domains.

### 3.5 Author contribution and co-citation analysis

Author-level indicators were calculated from the WoSCC corpus using NP, NC, TLS, and H-index (**Table 4**). Eight of the ten listed authors had five publications, while two had four publications. Among the listed authors, Baihong Pan had the highest NC (265), Monowar Aziz had the highest H-index (7), and Jie Zhang had the highest TLS (59). Yongqing Li and Baihong Pan each had TLS = 37. These measures identify different dimensions of publication output, citation impact, and collaboration.

**Table 4:** Authors in the top 10 publications.

Rank	Author	NP	NC	Total Link Strength	H-index
1	aziz, monowar	5	170	16	7
2	li, yongqing	5	230	37	6
3	pan, baihong	5	265	37	5
4	sharma, atul	5	134	15	3
5	sharma, jyotika	5	134	15	5
6	thorlacius, henrik	5	193	20	5
7	wang, ping	5	170	16	5
8	zhang, jie	5	41	59	3
9	cichon, iwona	4	103	8	4
10	kolaczkowska, elzbieta	4	103	8	4

The author collaboration network (**Figure 6A**) contained several team-based clusters. The co-citation network (**Figure 6B**) highlighted foundational authors associated with the development of NET biology, neutrophil function, intravascular NET formation, and thromboinflammation.

**Figure 6:**
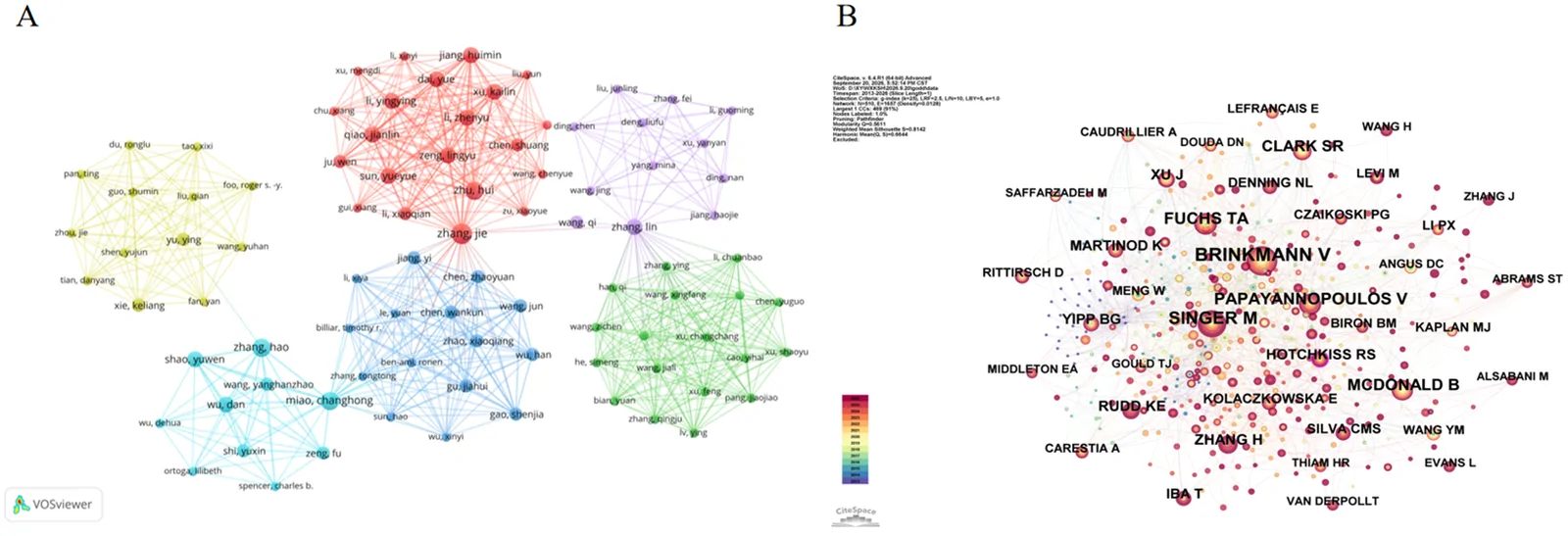
Author collaboration and co-citation networks. **(A)** Author co-occurrence network illustrating collaborative relationships. **(B)** Author co-citation network. Node size is proportional to author co-citation frequency. Larger nodes therefore represent authors who were more frequently co-cited within the analyzed corpus.

The co-citation structure therefore linked early NET-related mechanistic work with later studies of vascular injury, inflammation, and sepsis-associated organ dysfunction.

### 3.6 Co-cited references and cluster analysis

**Table 5** reports the top 10 co-cited references in the analyzed WoSCC corpus. The reported counts represent co-citation frequency within the analyzed corpus rather than global citation counts for the individual references. The most frequently co-cited reference was the 2023 review on neutrophils, NETs, and endothelial cell dysfunction in sepsis (33 co-citations). It was followed by the 2021 study of gasdermin D inhibition and NET formation in sepsis (31), the 2017 study of platelet-NET cooperation in intravascular coagulation (27), and the 2018 review by Papayannopoulos on NETs in immunity and disease (23). Four additional references each had 18 or fewer co-citations and addressed ferroptosis-associated lung injury, CXCR1/2-mediated NETosis, STING-associated immunothrombosis, DAMPs and NETs, infant sepsis, or pathogen-induced lung injury.

**Table 5:** The top 10 co-cited references in the analyzed corpus.

Rank	Year	Article	Total Citations	IF	Type of Study
1	2023	Neutrophil, neutrophil extracellular traps and endothelial cell dysfunction in sepsis. (PMID: 36629024)	33	7.9	Review
2	2021	Gasdermin D inhibition prevents multiple organ dysfunction during sepsis by blocking NET formation. (PMID: 36629024)	31	23.9	Article
3	2017	Platelets and neutrophil extracellular traps collaborate to promote intravascular coagulation during sepsis in mice. (PMID: 28073784)	27	23.9	Article
4	2018	Neutrophil extracellular traps in immunity and disease. (PMID:28990587 )	23	47.1	Review
5	2022	Neutrophil extracellular traps mediate m6A modification and regulates sepsis-associated acute lung injury by activating ferroptosis in alveolar epithelial cells. (PMID: 35637949)	18	11.7	Article
6	2022	Reduction of NETosis by targeting CXCR1/2 reduces thrombosis, lung injury, and mortality in experimental human and murine sepsis. (PMID: 34893315)	18	10.3	Article
7	2023	Neutrophil extracellular traps contribute to immunothrombosis formation via the STING pathway in sepsis-associated lung injury. (PMID: 37626060)	18	10.4	Article
8	2019	DAMPs and NETs in Sepsis. (PMID: 31736963)	17	7.0	Review
9	2019	Neutrophil extracellular traps (NETs) exacerbate severity of infant sepsis. (PMID: 30961634)	14	11.4	Article
10	2018	Maladaptive role of neutrophil extracellular traps in pathogen-induced lung injury. (PMID: 29415887)	12	6.8	Article

The reference co-citation network (**Figure 7A**) illustrates the intellectual foundation of NET-related sepsis research. In this network, node size represents citation frequency, and links indicate co-citation relationships. Several landmark references occupied central positions, including Singer M (2016), McDonald B (2017), Papayannopoulos V (2018), Thiam HR (2020), Rudd KE (2020), Zhang H (2023), Wiersinga WJ (2022), and Zhang J (2025). The prominent presence of Singer M (2016), which introduced the Sepsis-3 consensus definition, indicates that the redefinition of sepsis as a dysregulated host response has fundamentally shaped the research landscape. Papayannopoulos V (2018) provided a comprehensive framework for understanding NETs in immunity and disease, while McDonald B (2017) and Thiam HR (2020) contributed mechanistic insights into platelet-NET interactions and the biophysical regulation of NET formation.

**Figure 7:**
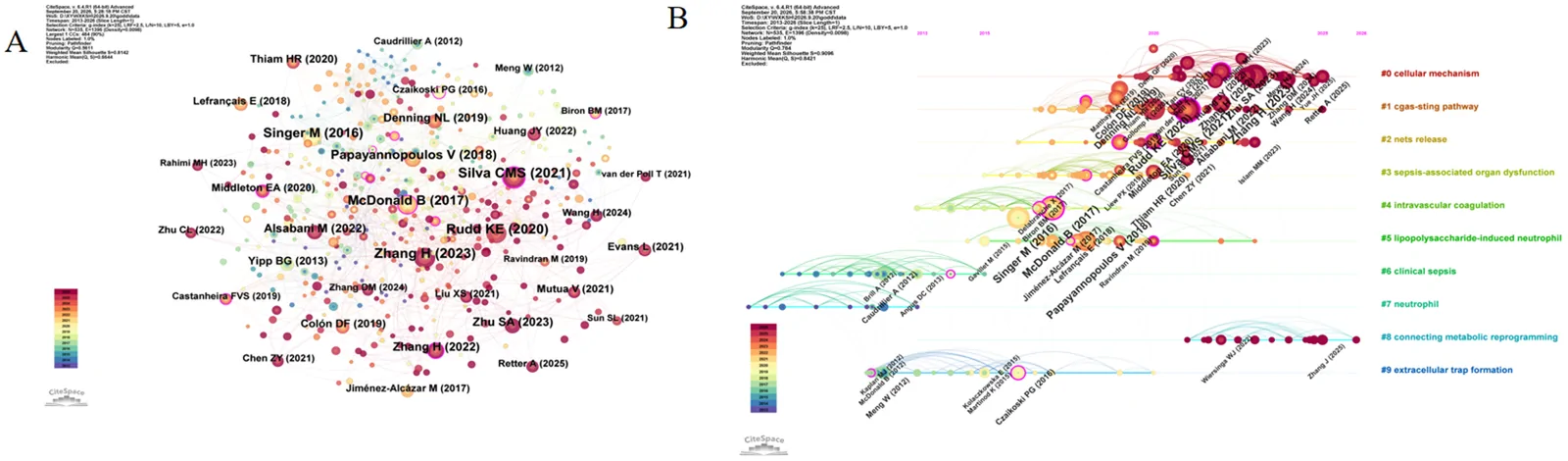
Co-citation structure, temporal evolution, and citation bursts of references. **(A)** Co-citation network of references. Links indicate co-citations, and node size represents citation frequency. **(B)** Timeline map of reference co-citation clusters. The horizontal axis spans from 2013 to 2026, illustrating the 10 major research clusters. The 2026 data are incomplete (search completed on September 19, 2026).

The timeline map of reference co-citation clusters (**Figure 7B**) revealed the temporal evolution of 10 major research themes (#0 to #9). These clusters were labeled as follows: #0 cellular mechanism, #1 cgas-sting pathway, #2 nets release, #3 sepsis-associated organ dysfunction, #4 intravascular coagulation, #5 lipopolysaccharide-induced neutrophil, #6 clinical sepsis, #7 neutrophil, #8 connecting metabolic reprogramming, and #9 extracellular trap formation. The horizontal extension of each cluster indicates the active period of its constituent references.

During the early period from 2013 to 2015, the timeline was dominated by clusters #9 extracellular trap formation, #7 neutrophil, and #6 clinical sepsis. This phase focused on establishing the basic biology of NET formation and the role of neutrophils in host defense. Cluster #9 encompassed early studies on the structural and functional characterization of NETs, while cluster #7 included research on neutrophil activation, phagocytosis, and antimicrobial mechanisms. Cluster #6 clinical sepsis began to consolidate the clinical definition of sepsis as a systemic inflammatory response. Together, these clusters represent the conceptual origins of the field, linking neutrophil biology to the clinical syndrome of sepsis.

From 2015 to 2020, the research emphasis expanded toward clusters #4 intravascular coagulation, #3 sepsis-associated organ dysfunction, and #2 nets release. The emergence of cluster #4 was marked by key nodes such as Singer M (2016), McDonald B (2017), and Papayannopoulos V (2018). Singer M (2016) introduced the Sepsis-3 consensus, redefining sepsis as life-threatening organ dysfunction caused by a dysregulated host response. McDonald B (2017) demonstrated that platelets and NETs collaborate to promote intravascular coagulation during sepsis, providing mechanistic evidence for the link between innate immunity and thrombosis. Papayannopoulos V (2018) offered a comprehensive review of NETs in immunity and disease, synthesizing the dual protective and injurious roles of NETs. Cluster #3, represented by Thiam HR (2020), focused on the biophysical mechanisms of NET formation and their contribution to organ injury. Cluster #2 nets release began to emerge, reflecting growing interest in the regulatory pathways controlling NET release. This phase marked a transition from descriptive NET biology to mechanistic studies of immunothrombosis and organ dysfunction.

From 2020 to 2025, the field deepened its mechanistic understanding, as reflected by clusters #0 cellular mechanism, #1 cgas-sting pathway, and continued activity in #2 nets release and #8 connecting metabolic reprogramming. Cluster #0 included studies on the cellular processes regulating NET formation, such as autophagy, cell death, and intracellular signaling. Cluster #1, represented by Zhang H (2023), highlighted the emerging role of innate immune sensing pathways in NET-related sepsis. The cGAS-STING axis, known for detecting cytosolic DNA, has been increasingly implicated in inflammatory responses and may link NET-derived DNA to sustained immune activation. Cluster #2 remained active, with studies focusing on the molecular regulators of NETosis, including PAD4 and histone citrullination. Cluster #8, represented by Wiersinga WJ (2022), suggests that immunometabolism is becoming an important dimension in NET research. Metabolic reprogramming of neutrophils, including glycolysis and oxidative phosphorylation, may influence their capacity to form NETs and their functional heterogeneity. This period reflects a shift toward specific signaling cascades, cellular processes, and metabolic regulation.

In the most recent period from 2025 to 2026, although limited to a short time window, cluster #8 connecting metabolic reprogramming remained prominent, represented by Zhang J (2025). This suggests that metabolic regulation of neutrophils continues to be an active area of investigation. The emergence of this node in the most recent period indicates that researchers are increasingly focusing on how metabolic pathways influence NET formation and neutrophil functional heterogeneity. This trend aligns with the broader shift toward understanding the context-dependent and stimulus-specific mechanisms that drive NET-related pathology in sepsis.

Taken together, the co-citation network and timeline analysis demonstrate that NET-related sepsis research has evolved from early descriptive studies of NET formation and clinical sepsis recognition toward a more mechanistic and clinically oriented field. The knowledge base has expanded from fundamental neutrophil biology to include intravascular coagulation, cGAS-STING signaling, metabolic reprogramming, and stimulus-specific neutrophil activation. This trajectory suggests that the field is maturing, with an increasing emphasis on understanding the heterogeneity of NET biology and its translation into clinical applications.

### 3.7 Author-keyword hotspots and thematic evolution

Keyword analyses used author keywords only: the WoSCC DE field after thesaurus cleaning and the Scopus Author Keywords field. In WoSCC, the most frequent terms were sepsis (180 occurrences; TLS = 915), neutrophil extracellular traps (146; TLS = 739), NETosis (72; TLS = 435), inflammation (63; TLS = 370), neutrophil (61; TLS = 353), activation (50; TLS = 281), platelet (34; TLS = 225), acute lung injury (29; TLS = 151), septic shock (28; TLS = 147), and infection (27; TLS = 172; **Table 6**).

**Table 6:** Top 10 keywords with the highest number of occurrences.

Rank	Keyword	Occurrences	Total Link Strength
1	sepsis	180	915
2	neutrophil extracellular traps (nets)	146	739
3	netosis	72	435
4	inflammation	63	370
5	neutrophil	61	353
6	activation	50	281
7	platelet	34	225
8	acute lung injury	29	151
9	septic shock	28	147
10	infection	27	172

The cleaned keyword network (**Figure 8A**) was organized around several highly interconnected hubs, including sepsis, neutrophil extracellular traps, NETosis, inflammation, neutrophil, platelet, and acute lung injury. These terms formed a dense central structure linking innate immunity, coagulation, and organ injury, with peripheral nodes representing specific mechanisms such as pyroptosis, autophagy, gasdermin D, and cell-free DNA. The keyword clustering analysis (**Figure 8B**) generated 10 major clusters: #0 cell death, #1 lysine lactylation, #2 autophagy, #3 vascular dysfunction, #4 binge drinking, #5 neutrophil extracellular trap, #6 septic shock, #7 migration, #8 TRPM2, and #9 tissue damage. These clusters encompassed core pathophysiological processes, critical care complications, and emerging molecular targets.

**Figure 8:**
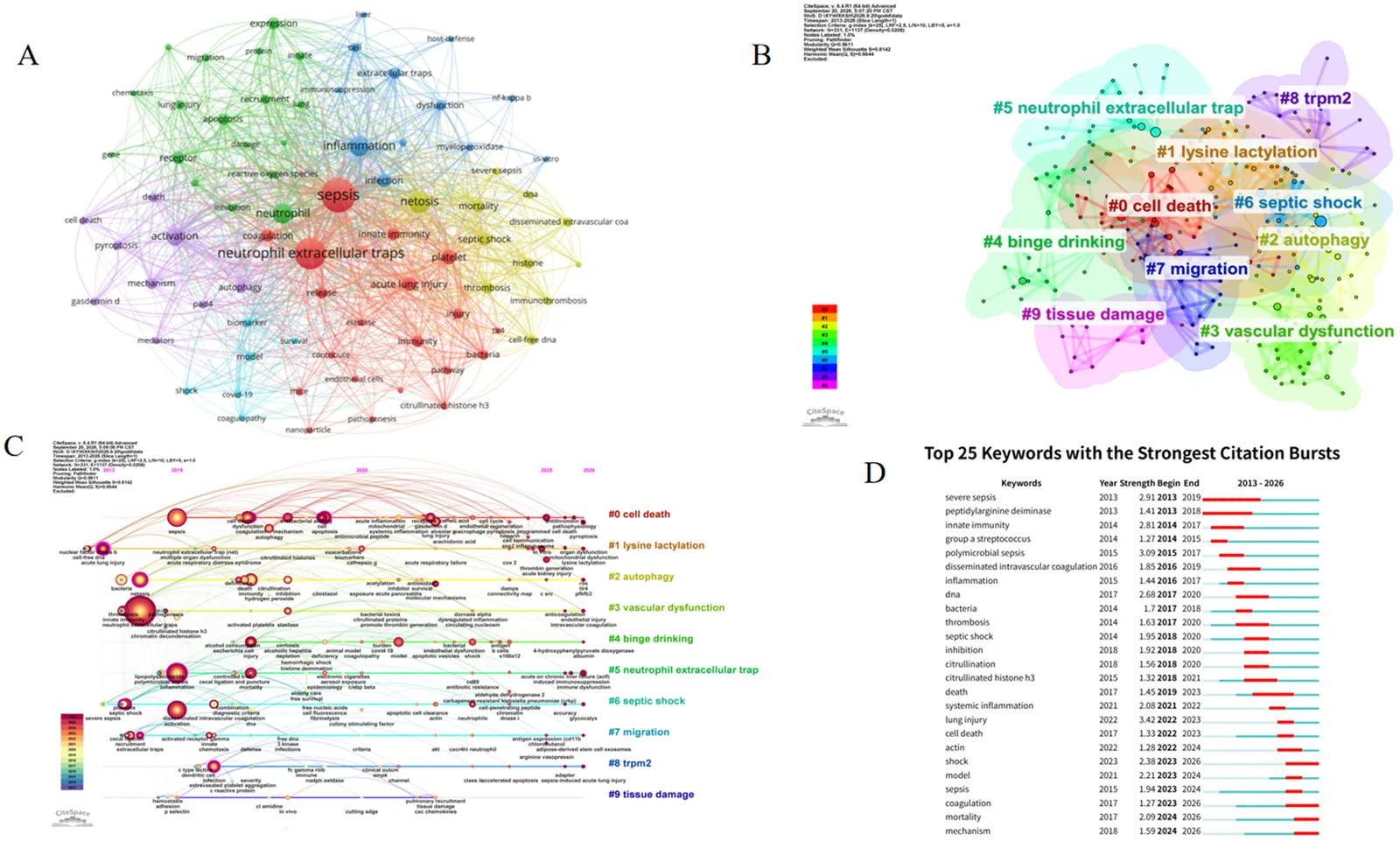
Keyword clustering and temporal evolution of research hotspots. **(A)** Keyword co-occurrence network. **(B)** Keyword clustering analysis demonstrating the main research topics in this field. Each cluster is represented by a distinct color and labeled with its most representative keyword. **(C)** Keyword clustering timeline map. Each colored line represents a clustered research theme, with its horizontal extension indicating the temporal span of the theme. **(D)** Top 25 keywords with the strongest citation bursts. The red segments indicate the burst duration, reflecting temporal changes in research attention.

The keyword cluster timeline (**Figure 8C**) revealed a clear temporal evolution of research hotspots from 2013 to 2026. During the early period (2013–2015), the dominant themes were centered on core sepsis and NET-related concepts, including sepsis, neutrophil extracellular traps, NETosis, inflammation, acute lung injury, and septic shock, corresponding to clusters #5 neutrophil extracellular trap, #6 septic shock, and #9 tissue damage. From 2015 to 2020, the research focus expanded toward vascular dysfunction, thrombosis, coagulation, autophagy, and cell death, with increasing activity in clusters #3 vascular dysfunction, #2 autophagy, and #0 cell death. Between 2020 and 2025, emerging themes included lysine lactylation, migration, TRPM2, ferroptosis, and pyroptosis, reflecting clusters #1 lysine lactylation, #7 migration, and #8 TRPM2. In the most recent period (2025–2026), the timeline continued to show keywords related to clinical outcomes and underlying mechanisms, indicating sustained attention to the translational and mechanistic aspects of NET-related sepsis research. These clusters describe changing research attention and do not by themselves establish mechanistic or clinical importance.

Keyword burst analysis can capture surges in academic attention within specific periods and serves as a practical tool for exploring emerging research frontiers. **Figure 8D** lists the top 25 keywords with the strongest citation bursts. Among them, “lung injury” exhibited the highest burst strength (3.42), followed by “polymicrobial sepsis” (3.09) and “severe sepsis” (2.91). In terms of mechanistic and disease exploration, early bursts were dominated by “severe sepsis,” “peptidylarginine deiminase,” “innate immunity,” “polymicrobial sepsis,” “disseminated intravascular coagulation,” “inflammation,” “DNA,” “bacteria,” “thrombosis,” and “septic shock,” reflecting the field’s initial focus on sepsis definitions, NET formation mechanisms, and coagulation abnormalities. In more recent years, bursts shifted toward “lung injury,” “cell death,” “systemic inflammation,” “actin,” “shock,” “model,” “sepsis,” “coagulation,” “mortality,” and “mechanism.” Critically, the keywords that continue to burst into 2026 mainly include “shock,” “coagulation,” “mortality,” and “mechanism.” This trend indicates that the latest research frontiers in NET-related sepsis have shifted toward clinical outcomes and unresolved pathophysiological mechanisms, with researchers committed to improving prognostic assessment and clarifying the mechanistic drivers of disease progression.

Taken together, the keyword analysis indicates that NET-related sepsis research has moved from early descriptive and mechanistic themes toward a broader and more clinically oriented landscape. The persistent bursts of shock, coagulation, mortality, and mechanism into 2026 suggest that core pathophysiological questions remain unresolved, while the emergence of lysine lactylation and TRPM2 points to new molecular directions that may shape future research.

### 3.8 Cross-database concordance assessment

Annual publication counts from WoSCC and Scopus showed a similar increasing pattern during the complete common period of 2013-2025 (**Figure 2C**). The Spearman rank correlation coefficient was rho = 0.986. The 2026 observations were incomplete and were excluded from the concordance calculation.

Among the top 20 author keywords, 12 were shared by the two databases, with 8 unique terms in each database (**Figure 9**, **Supplementary Table S4**). The Jaccard similarity coefficient was 0.429, indicating partial thematic overlap. Shared terms included sepsis, neutrophil extracellular traps, NETosis, inflammation, neutrophil, platelet, acute lung injury, septic shock, infection, coagulation, innate immunity, and apoptosis. Scopus-only terms included disseminated intravascular coagulation, pyroptosis, biomarker, acute respiratory distress syndrome, cell-free DNA, coagulopathy, immunothrombosis, and reactive oxygen species.

**Figure 9:**
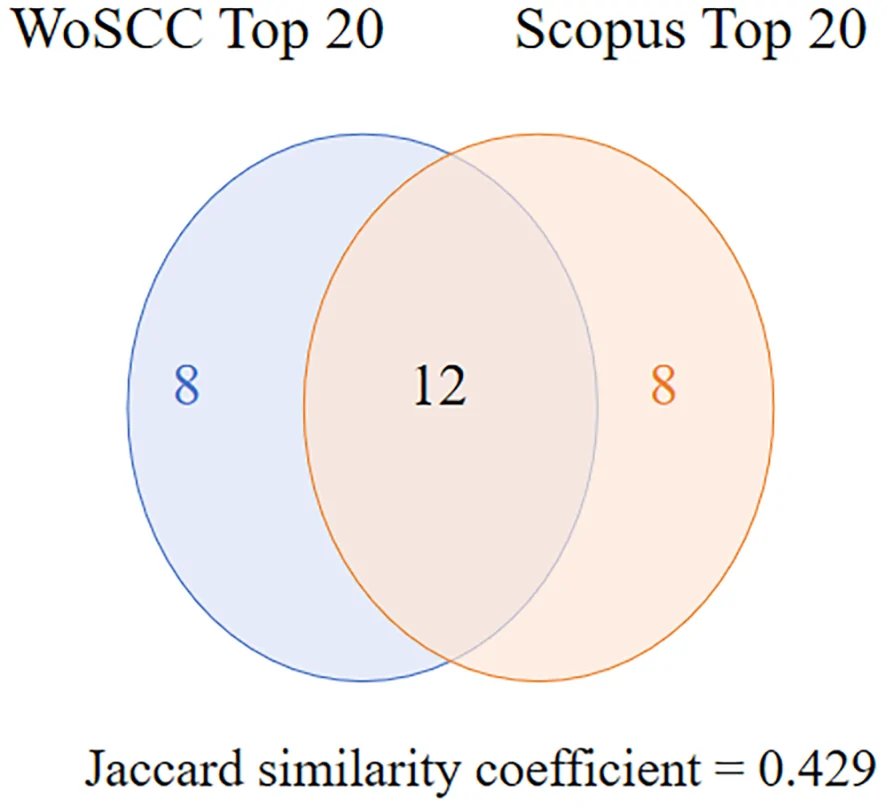
Venn diagram of the top 20 author keywords in WoSCC and Scopus. A total of 12 keywords overlapped between the two databases, with 8 unique keywords in each database. The Jaccard similarity coefficient was 0.429, indicating partial thematic overlap between the two top-20 keyword sets. WoSCC, Web of Science Core Collection.

In addition, the Scopus-based keyword, country, and institution networks showed several broadly similar thematic and collaboration patterns to those observed in the WoSCC analysis (**Supplementary Figures S1**-**S4**). These analyses were interpreted as a cross-database comparison between independently derived datasets rather than as validation based on a merged corpus.

## 4 Discussion

### 4.1 Growth of NET-related sepsis research and its scientific context

This study used independent WoSCC and Scopus corpora to characterize NET-related sepsis research. Publication output increased across the observed periods, and the complete common years showed strong concordance in annual trends (ρ = 0.986). The database-specific design avoids combining incompatible citation ecosystems and provides a clearer basis for interpreting publication output, collaboration, co-citation, and author-keyword patterns.

The observed increase in NET-related sepsis publications occurred alongside important conceptual developments in sepsis biology. One relevant development was the evolving framework of sepsis pathophysiology following the introduction of Sepsis-3, which emphasized the importance of dysregulated host responses and organ dysfunction (1). This conceptual transition has shifted the research emphasis from pathogen clearance toward host immune regulation, encompassing complex processes such as dysregulation of inflammatory networks, aberrant immune cell activation, and immune-coagulation interplay. Within this revised disease framework, NETs have gradually emerged as a key research focus for understanding the mechanisms of immune dysregulation in sepsis, owing to their capacity to bridge innate immune activation, inflammatory amplification, and tissue injury.

The conceptualization of NETs further reshaped the traditional understanding of neutrophil function. Brinkmann et al. first discovered that activated neutrophils release web-like structures composed of extracellular DNA and antimicrobial proteins, which participate in pathogen capture and the limitation of infection spread. This finding broke through the conventional view of neutrophils primarily as phagocytic and bactericidal effector cells, redefining them as important immune cells capable of actively modulating the immune microenvironment (4). Subsequent research further demonstrated that NETs are involved not only in host defense against infection but also in inflammatory regulation, vascular responses, and tissue damage, thereby extending NETs research from purely antimicrobial mechanisms to infection-associated immunopathological processes (25). The dual nature of NETs, possessing both protective and injurious properties, provides a theoretical foundation for their emergence as an important entry point in sepsis research.

The bibliometric results indicate that recent research attention has expanded toward immunothrombosis, organ injury, and clinically oriented topics. This trend is closely tied to the development of the immunothrombosis theory. The traditional view holds that the coagulation system is primarily responsible for maintaining hemostatic balance, but its sustained or excessive activation can lead to microcirculatory disturbances and tissue damage (7). As key mediators linking immune activation and coagulation responses, NETs have further advanced the understanding of the interrelationship among infection, inflammation, and coagulation abnormalities (8). Consequently, the development of immunothrombosis theory has broadened the research value of NETs in sepsis and propelled the field from single-cell mechanistic studies toward systemic pathological network investigations.

In recent years, COVID-19-related immunothrombosis research has further heightened attention to NETs in severe infectious diseases. Although COVID-19 is not equivalent to traditional bacterial sepsis, both conditions share common pathological features, including infection-induced immune dysregulation, excessive inflammatory activation, and coagulation abnormalities. Middleton et al. found that patients with COVID-19-associated acute respiratory distress syndrome exhibit pronounced NET formation, which participates in pulmonary vascular immunothrombotic processes, providing crucial evidence for understanding NETs-mediated infection-related vascular injury (26). The updated burst profile emphasized innate immunity, thrombosis, citrullination, coagulation, lung injury, and cell death, indicating expanding attention to infection-associated vascular and tissue injury.

Taken together, the observed publication growth occurred in the context of several major developments in the field, including evolving concepts of sepsis pathophysiology, expanded understanding of NET biology, increasing interest in immunothrombosis, and growing attention to clinically oriented questions. These contextual developments may help explain the diversification of research themes from NET formation mechanisms toward immune regulation, vascular injury, disease stratification, and other patient-oriented topics.

### 4.2 Global research landscape and characteristics of international collaboration

Country, institution, and collaboration analyses showed a geographically concentrated research landscape. In WoSCC, China had the highest publication output, whereas the United States had the highest listed citation impact and collaboration strength. These results describe different bibliometric dimensions and should not be reduced to a simple quantity-versus-quality comparison.

The institutional results showed that Chinese institutions occupied eight of the ten listed positions. Central South University had the highest publication output and collaboration strength among the listed institutions, while the University of Michigan had the highest citation count and H-index. This pattern indicates strong institutional concentration with heterogeneous citation impact.

The country and institution networks also showed regional clustering. Chinese institutions formed dense domestic groups, while the United States and selected European institutions occupied connected positions in the international network. The observed structure supports continued attention to cross-regional collaboration, but the network metrics do not measure the scientific quality of individual studies.

At the journal level, *Frontiers in Immunology* was the most productive listed venue and had the highest listed WoSCC citation and collaboration indicators. Because JIF is a journal-level indicator, it should be interpreted as contextual information about the journal rather than as an article-level quality measure.

The author results further showed that publication output, citation counts, H-index, and collaboration strength highlighted different researchers depending on the bibliometric indicator considered. This divergence illustrates why no single bibliometric indicator can summarize the contribution or validity of a researcher’s work.

Overall, the database-specific analysis supports a concentrated but internationally connected field, with clear differences between productivity, citation impact, and collaboration structure.

### 4.3 Knowledge base and theoretical shift in NET-Related sepsis research: from NET formation mechanisms to systemic immunopathological networks

Document co-citation analysis can reveal the intellectual knowledge base and the process of theoretical evolution within a research field. Through author co-citation and reference co-citation analyses, this study finds that the knowledge structure of NET-related sepsis research has centered around several major conceptual themes, including the discovery of NET formation mechanisms, the expansion of NET functional roles, and the increasing integration of immunothrombosis-related concepts into sepsis research. Authors including Papayannopoulos V, Brinkmann V, Singer M, Fuchs TA, and Mcdonald B showed high co-citation prominence within the analyzed network, suggesting that their publications represent frequently referenced knowledge sources contributing to the development of NET-related sepsis research.

Early research primarily focused on elucidating the existence of NETs and their biological significance in host defense. In 2004, Brinkmann et al. first reported that activated neutrophils release extracellular web-like structures composed of DNA and antimicrobial proteins, proposing a novel immune defense mode in which NETs participate in pathogen capture and the limitation of infection spread (4). This discovery broke through the traditional understanding that neutrophils exert their antimicrobial effects primarily through phagocytosis and oxidative burst, leading to their redefinition as important effector cells capable of actively modulating the immune microenvironment. Subsequently, Wang et al. further demonstrated that histone citrullination plays a critical role in chromatin decondensation and NET formation, providing an important foundation for explaining the molecular mechanisms underlying NETosis (27).

As research deepened, NETs studies gradually shifted from “exploration of formation mechanisms” toward “elucidation of functional roles.” Papayannopoulos’s review was frequently co-cited within the analyzed corpus, indicating that it represents a commonly referenced source in the intellectual background of this literature (25). Existing studies have demonstrated that NETs are not simply protective antimicrobial structures, but rather a form of immune response highly dependent on the disease context and host status. During early infection, NETs can exert a host defense role by limiting pathogen dissemination; however, under conditions of persistent inflammation or dysregulated immune balance, excessive NET formation may lead to inflammatory amplification, endothelial injury, and tissue dysfunction. Consequently, NETs research has progressively expanded from a sole focus on antimicrobial functions to the study of infection-associated immune injury mechanisms.

This study further finds that literature related to immunothrombosis theory occupies an important position in the co-citation network, suggesting that the link between NET research and the pathological processes of sepsis has been established primarily through immune-coagulation interactions. The immunothrombosis theory proposed by Engelmann and Massberg emphasizes that the coagulation system participates not only in hemostasis but also constitutes an integral component of innate immune defense (7). Fuchs et al. also demonstrated that extracellular DNA structures released by NETs can promote thrombus formation, further revealing the important role of NETs in linking immune activation and coagulation abnormalities (8). These studies have prompted a reconceptualization of the pathological mechanisms of sepsis, recognizing that infection-induced injury does not originate from a single inflammatory response but rather from a complex network process driven jointly by immune activation, coagulation dysregulation, and vascular dysfunction.

Furthermore, the co-citation results of this study indicate that the evolution of the sepsis theoretical framework is also closely linked to the development of NET research. The Sepsis-3 definition proposed by Singer et al. emphasizes that the core mechanism of sepsis lies in a dysregulated host response to infection and subsequent organ dysfunction, rather than direct damage caused by the pathogen itself (1). This conceptual shift has provided a new disease context for NET research, directing greater attention toward host immune status and the process of immune dysregulation, and has also promoted the conceptual transition of NETs from a mere inflammatory accompanying indicator toward a potential disease-modulating factor.

Taken together, the co-citation analysis results of this study suggest that the co-citation and thematic patterns are consistent with a broadening of the field from early studies of NET formation and host defense toward research on inflammation, vascular dysfunction, immunothrombosis, and organ injury. Early studies established the theoretical basis for NETs as an innate immune defense mechanism; subsequent research further revealed the complex roles of NETs in inflammatory regulation and tissue injury; and in recent years, with the development of immunothrombosis theory, NETs have gradually emerged as a critical regulatory node connecting infection, immunity, and coagulation abnormalities. Collectively, these theoretical evolutions constitute the current knowledge framework for NET-related sepsis research and explain the sustained research momentum witnessed in this field in recent years.

### 4.4 Current research hotspots and clinical oriented themes: from mechanism discovery to disease stratification

Cleaned author-keyword and burst analyses indicate that research attention has expanded from core NET formation and sepsis terms toward vascular dysfunction, thrombosis, cell death, autophagy, tissue injury, and disease-stratification themes. Because these signals reflect publication attention, they should be interpreted as thematic tendencies rather than as evidence of clinical translation.

#### 4.4.1 Immunothrombosis as an emerging hotspot in NET-related sepsis research

The cleaned keyword and burst results repeatedly included thrombosis, coagulation, disseminated intravascular coagulation, and immunothrombosis-related terms. This pattern is consistent with sustained interest in the interface between innate immunity and coagulation, but bibliometric co-occurrence cannot establish the direction or magnitude of biological effects.

Traditional sepsis research has often regarded coagulation disorders as secondary changes resulting from the inflammatory response, whereas the development of immunothrombosis theory emphasizes that the coagulation process itself is also an integral component of innate immune defense (7). Within this theoretical framework, NETs are considered a key factor linking neutrophil activation, platelet responses, and coagulation regulation (8).

However, the current research emphasis is no longer simply on demonstrating whether NETs participate in immunothrombosis, but rather on further elucidating their differential roles across distinct disease stages. Moderate NET formation may help limit pathogen dissemination, whereas sustained or excessive NET release may promote microcirculatory disturbances and organ injury. Therefore, the advancement of immunothrombosis research reflects a deeper understanding within the field regarding the functional complexity of NETs.

#### 4.4.2 NET-related biomarker research reflects the demand for disease stratification

The cross-database comparison identified biomarker among the Scopus-only high-frequency terms, while mortality and risk-related terms appeared in the WoSCC burst profile. These signals indicate attention to disease stratification, but they do not establish the diagnostic performance of any NET-related marker.

Sepsis patients exhibit a high degree of clinical heterogeneity. Although traditional inflammatory markers can reflect certain aspects of disease status, they are often insufficient for fully characterizing the complex immunological changes occurring in patients. NETs originate from extracellular chromatin structures released by activated neutrophils, and their associated molecular components may provide information distinct from that offered by conventional indicators, thereby rendering them increasingly important targets of investigation in infection immunology research (28, 29). Currently, indicators used to evaluate NETs formation primarily include circulating cell-free DNA (cfDNA), myeloperoxidase-DNA complexes (MPO-DNA complexes), neutrophil elastase-DNA complexes (NE-DNA complexes), and citrullinated histone H3 (CitH3), among others. However, different NETs indicators reflect distinct biological steps within the NETosis process, and their clinical interpretation remains subject to variation. For example, cfDNA is derived not only from NETosis but can also be generated by other forms of cell death, thus limiting its specificity; in contrast, MPO-DNA complexes and CitH3 are more reflective of processes associated with NETs formation, yet their detection methods and clinical interpretation still lack unified standards (30). Therefore, the increasing appearance of biomarker-related terms suggests growing research attention to the potential use of NET-associated measurements for patient characterization and disease stratification. Whether such markers provide clinically useful diagnostic or prognostic information requires dedicated validation studies.

#### 4.4.3 Risk and outcome themes in NET-related sepsis research

Terms related to mortality, risk assessment, models, and biomarkers appeared in the cleaned keyword or burst analyses. Their presence suggests interest in linking NET-related biology with patient stratification and outcome assessment. Such bibliometric signals do not demonstrate that a model or biomarker is clinically valid.

Risk-assessment studies may combine NET-related indicators with inflammatory cytokines, coagulation parameters, organ-function indices, and clinical variables. Any resulting model would require transparent reporting, external validation, calibration assessment, and evaluation of incremental value before clinical use.

#### 4.4.4 Therapeutic attention and NET-related sepsis research

The reference and keyword analyses included terms related to inhibition, citrullination, NETosis, and coagulation. These patterns indicate attention to regulatory and therapeutic concepts, but bibliometric prominence cannot establish therapeutic efficacy.

Mechanistic studies have examined NET degradation, NETosis regulation, PAD4-related chromatin decondensation, and NET-associated inflammatory or coagulation pathways. These topics provide a map of experimental attention rather than proof that any intervention improves outcomes in patients with sepsis.

However, given that NETs participate simultaneously in both host defense against infection and immune-mediated injury processes, the complete inhibition of NET formation may compromise host anti-infection capacity. Given the context-dependent roles of NETs in host defense and tissue injury, future experimental and clinical studies may examine whether selective modulation of excessive or persistent NET activity offers advantages over nonspecific suppression of NET formation.

#### 4.4.5 Evolution of research hotspots and translationally oriented research attention

Integrating co-occurrence, clustering, and burst results, the analysis identified three connected areas of attention: immunothrombosis and vascular dysfunction; disease stratification and outcome assessment; and experimental regulation of NET formation, clearance, or downstream injury. These categories organize the literature without implying that the field has established diagnostic or therapeutic solutions.

The central transition in the updated literature is therefore a broadening from NET formation mechanisms toward integrated studies of immunity, coagulation, vascular injury, organ damage, and patient-oriented assessment. The evidence remains bibliometric and should be interpreted within that boundary.

### 4.5 Limitations and future perspectives

This study employs bibliometric methods to analyze developmental trends, knowledge structure, and research attention in global NET-related sepsis research; several limitations should be acknowledged.

First, although WoSCC and Scopus were searched independently and analyzed separately, both databases may omit regional journals, non-English publications, and other document types. WoSCC served as the primary bibliometric source, and Scopus was used for independent concordance assessment; the two databases were not merged because they differ in journal coverage and citation-counting mechanisms. Second, the search strategy was restricted to established NET terminology (“Neutrophil Extracellular Trap,” “neutrophil extracellular DNA traps,” and “NETosis”) and excluded ambiguous terms such as “NET” and broad experimental terms such as “LPS” and “endotoxemia.” Although this improved specificity, relevant studies using alternative wording may have been missed, and only English-language articles and reviews were included. Third, citation-related indicators, including NC and H-index, were calculated only within WoSCC and were not combined with Scopus metrics. Citation counts are influenced by self-citation, disciplinary conventions, and database coverage, and the 2026 data are incomplete because the search was completed on September 19, 2026; 2026 was therefore not directly compared with complete years. Fourth, keyword analyses used only author keywords (DE field in WoSCC; Author Keywords in Scopus), and Keywords Plus and Index Keywords were excluded. A thesaurus file was applied to merge synonymous expressions and remove generic indexing terms, but residual inconsistencies or indexing-term artifacts may still affect co-occurrence, clustering, and burst detection. Finally, bibliometric indicators reflect research output and citation impact rather than research quality, mechanistic validity, or clinical efficacy. The identified hotspots and thematic trends should therefore be interpreted as research attention patterns, not as evidence of diagnostic or therapeutic effectiveness.

Future research may benefit from further characterization of the functional heterogeneity and temporal dynamics of NETs, greater standardization of NET measurement and reporting, and multicenter validation of NET-related biomarkers and predictive models. Integration of molecular data with well-defined clinical phenotypes may also help clarify the translational relevance of NET-associated signals. NET-targeted interventions require cautious evaluation in appropriately designed experimental and clinical studies, particularly given the potential balance between host defense, inflammatory injury, coagulation, and bleeding-related risks.

## 5 Conclusion

This study used separate WoSCC and Scopus analyses to characterize the development of NET-related sepsis research. The WoSCC corpus contained 263 screened records and the Scopus corpus contained 499 screened records. WoSCC analysis showed China had the highest publication output among the listed countries, whereas the United States had the highest listed citation impact and collaboration strength. Author-keyword and co-citation analyses showed a knowledge structure centered on sepsis, NETosis, inflammation, immunothrombosis, vascular dysfunction, organ injury, and cell death.

The high correlation in complete-year publication trends (Spearman’s ρ = 0.986), together with partial overlap in high-frequency author keywords, indicates that the two database-specific corpora provide related but non-identical views of the field. The results should be read as patterns of research attention, publication output, collaboration, and citation impact. They do not establish biomarker performance, prognostic utility, or therapeutic efficacy.

Future studies may benefit from standardized NET measurement and reporting, prospective multicenter validation of NET-related biomarkers and predictive models, transparent data processing, and appropriately designed clinical investigations. Such studies will be needed to determine whether themes receiving increasing research attention are associated with reproducible and clinically meaningful advances in sepsis research and care.

## Data Availability

The original contributions presented in the study are included in the article and supplementary material. Further inquiries can be directed to the corresponding author.
