# Supplementary material for: Global trends and emerging frontiers in neutrophil extracellular trap-related sepsis research: a bibliometric analysis
Source: Front Immunol. 2026 Oct 26;17:1954033. doi: 10.3389/fimmu.2026.1954033 (PMC13745503; doi:10.3389/fimmu.2026.1954033)
Supplement: Supplementary file 1 [file DataSheet1.docx]

**Supplementary Figure S1. Scopus-based keyword co-occurrence network.**
The network was generated independently from the Scopus dataset using VOSviewer. Node size corresponds to keyword frequency, and node color distinguishes different clusters. This analysis provides an independent Scopus-based comparison of the thematic structure observed in the WoSCC analysis.

**Supplementary Figure S2. Scopus-based author keyword clustering.**
Clusters were generated independently from the Scopus dataset. Each cluster is represented by a distinct color and labeled with its most representative keyword. The Scopus-based clustering showed broad thematic similarities to the WoSCC-based results, while database-specific differences were also present.

**Supplementary Figure S3. Scopus-based country collaboration network.**
The network was constructed independently from the Scopus dataset. Node size corresponds to publication volume, and line thickness denotes collaboration intensity. Node colors distinguish different clusters of collaboration.

**Supplementary Figure S4. Scopus-based institution collaboration network.**
The network was constructed independently from the Scopus dataset. Node size corresponds to publication volume, and line thickness denotes collaboration intensity. Node colors distinguish different clusters of collaboration. This analysis provides an independent Scopus-based comparison of institutional collaboration patterns observed in the WoSCC analysis.
