# Supplementary material for: Global trends and emerging frontiers in neutrophil extracellular trap-related sepsis research: a bibliometric analysis
Source: Front Immunol. 2026 Oct 26;17:1954033. doi: 10.3389/fimmu.2026.1954033 (PMC13745503; doi:10.3389/fimmu.2026.1954033)
Supplement: Supplementary file 2 [file Table1.docx]

**Supplementary Table S1. Thesaurus rules for author keyword cleaning in WoSCC.**

| **Label** | **Prior to consolidation** | **After the merger** | **Combination Principle** |
| --- | --- | --- | --- |
| neutrophil extracellular traps | neutrophil extracellular trap | neutrophil extracellular traps | ① |
| sepsis | neutrophil extracellular traps (nets) | neutrophil extracellular traps | ① |
| activation | nets | neutrophil extracellular traps | ⑤ |
| inflammation | net formation | netosis | ④ |
| netosis | extracellular trap formation | netosis | ④ |
| acute lung injury | acute lung injury (ali) | acute lung injury | ⑤ |
| septic shock | disseminated intravascular coagulation (dic) | disseminated intravascular coagulation | ⑤ |
| neutrophil extracellular trap | gsdmd | gasdermin d | ④ |
| coagulation | aki | acute kidney injury | ④ |
| mortality | cell free dna | cell-free dna | ⑨ |
| innate immunity | cell-free dna (cfdna) | cell-free dna | ⑨ |
| immunity | blood platelets | platelets | ④ |
| extracellular traps | dendritic cells | dendritic cell | ① |
| apoptosis | cells | cell | ① |
| dysfunction | mechanisms | mechanism | ① |
| infection | cecum ligation and puncture | cecal ligation and puncture | ④ |
| platelets | acetyl salicylic acid | acetylsalicylic acid | ④ |
| bacteria | vitamin c | ascorbic acid | ④ |
| injury | acute respiratory distress syndrome (ards) | acute respiratory distress syndrome | ④ |
| histones | nf kappa b | nuclear factor kappa b | ④ |
| thrombosis | nf-kappa b | nuclear factor kappa b | ⑤ |
| model | regulated cell death | programmed cell death | ④ |
| lung injury | inflammatory response | inflammation | ④ |
| mechanisms | damage | injury | ④ |
| autophagy | bacterial elimination | bacterial clearance | ④ |
| neutrophil extracellular traps (nets) | anti-bacterial effect | antibacterial activity | ⑨ |
| death | lps | lipopolysaccharide | ⑤ |
| cells | immune dysregulation | immune dysfunction | ④ |
| inhibition | expression | delete |  |
| recruitment | release | delete |  |
| cell death | receptor | delete |  |
| disseminated intravascular coagulation | migration | delete |  |
| gasdermin d | mediators | delete |  |
| damage | bone marrow | delete |  |
| myeloperoxidase | contribute | delete |  |
| polymicrobial sepsis | binding | delete |  |
| cecal ligation | pathway | delete |  |
| elastase | roles | delete |  |
| dna | association | delete |  |
| severe sepsis | arthritis | delete |  |
| shock | blood | delete |  |
| chemotaxis | clearance | delete |  |
| citrullinated histone h3 | pathways | delete |  |
| molecular mechanisms | contributes | delete |  |
| receptors | binge drinking | delete |  |
| definitions | liver | delete |  |
| thrombin generation | delivery | delete |  |
| cathepsin g | target | delete |  |
| platelet activation | genes | delete |  |
| neutrophils | kill | delete |  |
| acute kidney injury | burn injury | delete |  |
| systemic inflammation | capacity | delete |  |
| programmed cell death | acid | delete |  |
| in vitro | care | delete |  |
| mice | acquire | delete |  |
| endothelial cells | failure | delete |  |
| cell free dna | a(2) | delete |  |
| dna traps | isoform | delete |  |
| pathogenesis |  |  |  |
| blood platelets |  |  |  |
| innate |  |  |  |
| p selectin |  |  |  |
| immunosuppression |  |  |  |
| citrullination |  |  |  |
| extracellular trap formation |  |  |  |
| peptidylarginine deiminase |  |  |  |
| cell-free dna |  |  |  |
| actin |  |  |  |
| pyroptosis |  |  |  |
| alveolar macrophage pyroptosis |  |  |  |
| inflammasome activation |  |  |  |
| epidemiology |  |  |  |
| s100a12 |  |  |  |
| severity |  |  |  |
| group a streptococcus |  |  |  |
| deep vein thrombosis |  |  |  |
| biomarkers |  |  |  |
| adhesion |  |  |  |
| critically ill patients |  |  |  |
| cecal ligation and puncture |  |  |  |
| regulated cell death |  |  |  |
| acute lung injury (ali) |  |  |  |
| burden |  |  |  |
| colony stimulating factor |  |  |  |
| chromatin decondensation |  |  |  |
| acute myocardial infarction |  |  |  |
| endothelial injury |  |  |  |
| mitochondrial dna |  |  |  |
| inflammatory response |  |  |  |
| streptococcus pneumoniae |  |  |  |
| therapeutic strategies |  |  |  |
| tissue damage |  |  |  |
| survival |  |  |  |
| intravascular coagulation |  |  |  |
| c/ebp beta |  |  |  |
| host defense |  |  |  |
| mechanism |  |  |  |
| promote thrombin generation |  |  |  |
| inhibitor |  |  |  |
| nets |  |  |  |
| acute pancreatitis |  |  |  |
| gsdmd |  |  |  |
| in vivo |  |  |  |
| induced immunosuppression |  |  |  |
| b cells |  |  |  |
| pathophysiology |  |  |  |
| mitochondrial |  |  |  |
| deficiency |  |  |  |
| pulmonary recruitment |  |  |  |
| lipopolysaccharide |  |  |  |
| tlr4 |  |  |  |
| mesenchymal stem cells |  |  |  |
| glycocalyx |  |  |  |
| heparin |  |  |  |
| net formation |  |  |  |
| sepsis-induced acute lung injury |  |  |  |
| metabolic reprogramming |  |  |  |
| cell |  |  |  |
| nf-kappa b |  |  |  |
| nadph oxidase |  |  |  |
| mitochondrial dysfunction |  |  |  |
| lysine lactylation |  |  |  |
| cxc chemokines |  |  |  |
| immune dysfunction |  |  |  |
| pfkfb3 |  |  |  |
| organ dysfunction |  |  |  |
| immune dysregulation |  |  |  |
| global burden |  |  |  |
| ros |  |  |  |
| pad4 |  |  |  |
| ascorbic acid |  |  |  |
| cell biology |  |  |  |
| bacterial clearance |  |  |  |
| ciglitazone |  |  |  |
| 15 deoxy delta(12 |  |  |  |
| activated receptor gamma |  |  |  |
| bacterial elimination |  |  |  |
| kappa b |  |  |  |
| hemostasis |  |  |  |
| intensive care |  |  |  |
| anti-bacterial effect |  |  |  |
| dna cf dna/nets |  |  |  |
| leukocyte endothelial interactions |  |  |  |
| hes 130/04 |  |  |  |
| bacterial |  |  |  |
| anticoagulant therapy |  |  |  |
| critical illness |  |  |  |
| klebsiella infection |  |  |  |
| alkaline phosphatase |  |  |  |
| antiplatelet drugs |  |  |  |
| acetylsalicylic acid |  |  |  |
| cell-free dna (cfdna) |  |  |  |
| disseminated intravascular coagulation (dic) |  |  |  |
| cell fluorescence |  |  |  |
| cilostazol |  |  |  |
| acetyl salicylic acid |  |  |  |
| cirrhosis |  |  |  |
| atherosclerosis |  |  |  |
| elastase inhibitor |  |  |  |
| 5 lipoxygenase |  |  |  |
| inflammatory response syndrome |  |  |  |
| alcoholic hepatitis |  |  |  |
| clarithromycin |  |  |  |
| community acquired pneumonia |  |  |  |
| caffeic acid |  |  |  |
| depletion |  |  |  |
| extravasated platelet aggregation (epa) |  |  |  |
| lung |  |  |  |
| acute respiratory distress syndrome (ards) |  |  |  |
| anticoagulant activity |  |  |  |
| bacteremia |  |  |  |
| elderly care |  |  |  |
| polymorphisms |  |  |  |
| lps |  |  |  |
| murine macrophages |  |  |  |
| criteria |  |  |  |
| activated platelets |  |  |  |
| cell signaling |  |  |  |
| apoptotic cells |  |  |  |
| atp synthase |  |  |  |
| antigen |  |  |  |
| b-1a cells |  |  |  |
| adenosine triphosphate (atp) |  |  |  |
| diagnostic criteria |  |  |  |
| pulmonary |  |  |  |
| degradation |  |  |  |
| alpha |  |  |  |
| aim2 inflammasome |  |  |  |
| decondensation |  |  |  |
| antibacterial immunity |  |  |  |
| coagulopathy |  |  |  |
| defense |  |  |  |
| dendritic cells |  |  |  |
| antibiotic resistance |  |  |  |
| controlled trial |  |  |  |
| citrullinated histones |  |  |  |
| antimicrobial activity |  |  |  |
| c type lectins |  |  |  |
| acetylation |  |  |  |
| bacterial infection |  |  |  |
| granulopoiesis |  |  |  |
| 3d biomodels |  |  |  |
| ampk |  |  |  |
| caspases |  |  |  |
| adenosine |  |  |  |
| cystic fibrosis |  |  |  |
| endoplasmic reticulum stress |  |  |  |
| blood-brain barrier |  |  |  |
| cell communication |  |  |  |
| homeostasis |  |  |  |
| antigen expression (cd11b |  |  |  |
| dendritic cell |  |  |  |
| plasma dna |  |  |  |
| francisella tularensis |  |  |  |
| carbapenem-resistant klebsiella pneumoniae (crkp) |  |  |  |
| cell-penetrating peptide |  |  |  |
| chromatin |  |  |  |
| brain injury |  |  |  |
| 5-hydroxytryptophan (5-ht) |  |  |  |
| acute peritonitis |  |  |  |
| cutting edge |  |  |  |
| acute inflammation |  |  |  |
| respiratory distress syndrome |  |  |  |
| class ii |  |  |  |
| nuclear factor kappa b |  |  |  |
| 3 kinase |  |  |  |
| c reactive protein |  |  |  |
| microdomains |  |  |  |
| nf kappa b |  |  |  |
| transcriptional regulation |  |  |  |
| cardiovascular disease |  |  |  |
| channel |  |  |  |
| c src |  |  |  |
| cl amidine |  |  |  |
| arginine vasopressin |  |  |  |
| cardiac dysfunction |  |  |  |
| cox 2 |  |  |  |
| antithrombin |  |  |  |
| cell cycle |  |  |  |
| vitamin c |  |  |  |
| aldehyde dehydrogenase 2 |  |  |  |
| cd64 index on neutrophils |  |  |  |
| acute respiratory distress syndrome |  |  |  |
| kidney injury |  |  |  |
| cxcr4hi neutrophil |  |  |  |
| antibacterial activity |  |  |  |
| biomimetic nanoparticle |  |  |  |
| astragaloside iv |  |  |  |
| ccr2 |  |  |  |
| circulating nucleosm |  |  |  |
| akt |  |  |  |
| cerna network |  |  |  |
| acute respiratory failure |  |  |  |
| aki |  |  |  |
| apolipoprotein m |  |  |  |
| accelerated apoptosis |  |  |  |
| antimicrobial peptide |  |  |  |
| cd64 expression |  |  |  |
| complement c5a receptor 1 |  |  |  |
| cd89 |  |  |  |
| cell differentiation |  |  |  |
| covid 19 |  |  |  |
| bacterial toxins |  |  |  |
| cecum ligation and puncture |  |  |  |
| antioxidant |  |  |  |
| animal model |  |  |  |
| combination |  |  |  |
| aerosol exposure |  |  |  |
| 4-hydroxyphenylpyruvate dioxygenase |  |  |  |
| activated protein c |  |  |  |
| alcohol consumption |  |  |  |
| cholesterol biosynthesis |  |  |  |
| cd177 |  |  |  |
| arachidonic acid |  |  |  |
| bone marrow mesenchymal stem cells |  |  |  |
| diagnosis |  |  |  |
| connectivity map |  |  |  |

① = Consistency between singular and plural forms

② = Standardization of spelling variants

③ = Remove parenthetical abbreviations and retain the full names.

④ = Synonyms

⑤ = Consistency between abbreviations and full names

⑥ = Spelling correction

⑦ = Remove bracketed notes

⑧ = Standard interchangeable discipline names

⑨ = Standardization of hyphen usage

**Supplementary Table S2. Thesaurus rules for author keyword cleaning in Scopus.**

| **Label** | **Prior to consolidation** | **After the merger** | **Combination Principle** |
| --- | --- | --- | --- |
| sepsis | extracellular trap | extracellular traps | ① |
| extracellular trap | neutrophil extracellular trap | neutrophil extracellular traps | ① |
| neutrophil | neutrophile extracellular trap | neutrophil extracellular traps | ①+② |
| metabolism | neutrophil extracellular traps (nets) | neutrophil extracellular traps | ③ |
| extracellular traps | neutrophil | neutrophils | ① |
| neutrophils | macrophage | macrophages | ① |
| animal experiment | cytokine | cytokines | ① |
| animal model | histone | histones | ① |
| neutrophil extracellular trap | disease models | disease model | ① |
| inflammation | lipopolysaccharides | lipopolysaccharide | ① |
| immunology | drug effects | drug effect | ① |
| pathology | bacterial infections | bacterial infection | ① |
| neutrophil extracellular traps | blood vessels | blood vessel | ① |
| disease model | cell free nucleic acids | cell free nucleic acid | ① |
| disease models | thrombocyte | blood platelets | ④ |
| netosis | blood clotting | blood coagulation | ④ |
| interleukin 6 | blood clotting disorder | blood coagulation disorders | ①+④ |
| enzyme linked immunosorbent assay | disseminated intravascular clotting | disseminated intravascular coagulation | ④ |
| complication | acute kidney failure | acute kidney injury | ④ |
| tumor necrosis factor | adult respiratory distress syndrome | acute respiratory distress syndrome | ④ |
| genetics | alanine transaminase | alanine aminotransferase | ④ |
| in vitro study | endothelium cell | endothelial cells | ①+④ |
| priority journal | mortality rate | mortality | ④ |
| protein expression | survival rate | survival | ④ |
| blood | covid 19 | coronavirus disease 2019 | ⑤ |
| lipopolysaccharide | reactive oxygen specy | reactive oxygen metabolite | ④+⑥ |
| myeloperoxidase | autophagy (cellular) | autophagy | ⑦ |
| biological marker | bacterium | bacteria | ① |
| interleukin 1beta | bacteria (microorganisms) | bacteria | ⑦ |
| signal transduction | computational biology | bioinformatics | ⑧ |
| flow cytometry | protein arginine deiminase type 4 | peptidylarginine deiminase 4 | ④ |
| drug effect | padi2 protein | protein arginine deiminase type 2 | ④ |
| reactive oxygen metabolite | cell free dna | cell-free dna | ⑨ |
| cecal ligation and puncture induced sepsis | circulating free dna | cell-free dna | ④ |
| in vivo study | bacterial count | bacterial load | ④ |
| histone | biological marker | biomarkers | ① |
| innate immunity | endotoxin | lipopolysaccharide | ④ |
| immune response | tumor necrosis factor alpha | tumor necrosis factor | ④ |
| drug therapy | macromolecular substances | macromolecule | ① + ④ |
| western blotting | human | delete |  |
| acute lung injury | article | delete |  |
| apoptosis | nonhuman | delete |  |
| immunofluorescence | controlled study | delete |  |
| septic shock | animals | delete |  |
| disease severity | humans | delete |  |
| cytokine | animal | delete |  |
| macrophage | mouse | delete |  |
| survival rate | male | delete |  |
| gene expression | mice | delete |  |
| neutrophil chemotaxis | animal tissue | delete |  |
| lipopolysaccharides | animal cell | delete |  |
| pathophysiology | female | delete |  |
| histopathology | c57bl mouse | delete |  |
| physiology | unclassified drug | delete |  |
| etiology | adult | delete |  |
| phagocytosis | human cell | delete |  |
| multiple organ failure | review | delete |  |
| biomarkers | aged | delete |  |
| blood clotting | middle aged | delete |  |
| lung injury | upregulation | delete |  |
| real time polymerase chain reaction | major clinical study | delete |  |
| dna | clinical article | delete |  |
| leukocyte elastase | chemistry | delete |  |
| leukocyte activation | kegg | delete |  |
| mortality | gene | delete |  |
| blood sampling | clinical outcome | delete |  |
| histones | double stranded dna | delete |  |
| histone h3 | comparative study | delete |  |
| lung parenchyma | creatinine | delete |  |
| deoxyribonuclease i | bone marrow cell | delete |  |
| endothelium cell | escherichia coli | delete |  |
| protein arginine deiminase type 4 | rat | delete |  |
| gene ontology | coculture | delete |  |
| cytokines | human tissue | delete |  |
| therapy | rats | delete |  |
| lung | cells | delete |  |
| knockout mouse | diseases | delete |  |
| immunocompetent cell | apache | delete |  |
| immunohistochemistry | eosin | delete |  |
| thrombocyte | intestine flora | delete |  |
| pathogenesis | normal human | delete |  |
| blood clotting disorder | very elderly | delete |  |
| macrophages | cecum | delete |  |
| sepsis induced acute lung injury | c57bl 6 mouse | delete |  |
| cell death | bone marrow cells | delete |  |
| pyroptosis | arthritis | delete |  |
| disseminated intravascular clotting | bagg albino mouse | delete |  |
| reactive oxygen specy | liver | delete |  |
| cytokine production | asca | delete |  |
| oxidative stress | cancer growth | delete |  |
| diagnosis |  |  |  |
| microbiology |  |  |  |
| prospective study |  |  |  |
| thrombosis |  |  |  |
| sequential organ failure assessment score |  |  |  |
| rna sequencing |  |  |  |
| blood platelets |  |  |  |
| interleukin 10 |  |  |  |
| thromboinflammation |  |  |  |
| thrombocyte activation |  |  |  |
| interleukin 8 |  |  |  |
| bacterial load |  |  |  |
| bronchoalveolar lavage fluid |  |  |  |
| intensive care unit |  |  |  |
| enzyme activity |  |  |  |
| differential gene expression |  |  |  |
| pharmacology |  |  |  |
| protein function |  |  |  |
| disseminated intravascular coagulation |  |  |  |
| receptors |  |  |  |
| peroxidase |  |  |  |
| alanine aminotransferase |  |  |  |
| cohort analysis |  |  |  |
| tissue injury |  |  |  |
| immunofluorescence assay |  |  |  |
| protein protein interaction |  |  |  |
| deoxyribonuclease |  |  |  |
| neutrophil activation |  |  |  |
| sepsis associated coagulopathy |  |  |  |
| platelet count |  |  |  |
| bioinformatics |  |  |  |
| circulating free dna |  |  |  |
| prognosis |  |  |  |
| acute kidney failure |  |  |  |
| fluorescence intensity |  |  |  |
| survival |  |  |  |
| citrullination |  |  |  |
| cell culture |  |  |  |
| cell viability |  |  |  |
| pneumonia |  |  |  |
| immunity |  |  |  |
| extracellular |  |  |  |
| phenotype |  |  |  |
| bacterial clearance |  |  |  |
| receiver operating characteristic |  |  |  |
| drug mechanism |  |  |  |
| lung edema |  |  |  |
| endothelial dysfunction |  |  |  |
| procalcitonin |  |  |  |
| organ injury |  |  |  |
| mortality rate |  |  |  |
| high mobility group b1 protein |  |  |  |
| gene expression profiling |  |  |  |
| thrombin |  |  |  |
| hemostasis |  |  |  |
| adult respiratory distress syndrome |  |  |  |
| monocyte |  |  |  |
| immunosuppressive treatment |  |  |  |
| shock |  |  |  |
| ferroptosis |  |  |  |
| cell infiltration |  |  |  |
| antiinflammatory agent |  |  |  |
| blood coagulation |  |  |  |
| cell migration |  |  |  |
| cell activation |  |  |  |
| leukocyte |  |  |  |
| neutrophil extracellular traps (nets) |  |  |  |
| lactic acid |  |  |  |
| exosome |  |  |  |
| bactericidal activity |  |  |  |
| inflammasome |  |  |  |
| antiinflammatory activity |  |  |  |
| drug effects |  |  |  |
| observational study |  |  |  |
| fibrinolysis |  |  |  |
| dendritic cell |  |  |  |
| ex vivo study |  |  |  |
| cxcl2 chemokine |  |  |  |
| cytology |  |  |  |
| histology |  |  |  |
| arginine deiminase |  |  |  |
| enzyme inhibition |  |  |  |
| c reactive protein |  |  |  |
| liver injury |  |  |  |
| acute kidney injury |  |  |  |
| endothelium injury |  |  |  |
| cd4+ t lymphocyte |  |  |  |
| vascularization |  |  |  |
| immunomodulation |  |  |  |
| hyperinflammation |  |  |  |
| cell isolation |  |  |  |
| neutrophil function |  |  |  |
| leukocyte count |  |  |  |
| cell free nucleic acid |  |  |  |
| diagnostic test accuracy study |  |  |  |
| cxcl1 chemokine |  |  |  |
| gene set enrichment analysis |  |  |  |
| thrombocytopenia |  |  |  |
| least absolute shrinkage and selection operator |  |  |  |
| nucleosome |  |  |  |
| coronavirus disease 2019 |  |  |  |
| critically ill patient |  |  |  |
| thrombocyte aggregation |  |  |  |
| covid 19 |  |  |  |
| lactate dehydrogenase |  |  |  |
| disease association |  |  |  |
| polarization |  |  |  |
| quantitative analysis |  |  |  |
| protein |  |  |  |
| cell free dna |  |  |  |
| klebsiella pneumoniae |  |  |  |
| bacterial growth |  |  |  |
| machine learning |  |  |  |
| kidney injury |  |  |  |
| endotoxemia |  |  |  |
| antigens |  |  |  |
| isoflurane |  |  |  |
| neutrophil count |  |  |  |
| calgranulin b |  |  |  |
| endothelial cells |  |  |  |
| aspartate aminotransferase |  |  |  |
| blood coagulation disorders |  |  |  |
| peptidylarginine deiminase 4 |  |  |  |
| transcriptomics |  |  |  |
| regulated cell death |  |  |  |
| endotoxin |  |  |  |
| principal component analysis |  |  |  |
| hl 60 cell line |  |  |  |
| citrullinated histone h3 |  |  |  |
| immune dysregulation |  |  |  |
| molecular docking |  |  |  |
| correlation analysis |  |  |  |
| single cell rna seq |  |  |  |
| bacteria (microorganisms) |  |  |  |
| dna histone interaction |  |  |  |
| mitogen activated protein kinase |  |  |  |
| cell free nucleic acids |  |  |  |
| cell interaction |  |  |  |
| necroptosis |  |  |  |
| area under the curve |  |  |  |
| sensitivity and specificity |  |  |  |
| adaptive immunity |  |  |  |
| procedures |  |  |  |
| ascorbic acid |  |  |  |
| computational biology |  |  |  |
| complement activation |  |  |  |
| immune deficiency |  |  |  |
| plasminogen activator inhibitor 1 |  |  |  |
| rna sequence |  |  |  |
| umbilical vein endothelial cell |  |  |  |
| calgranulin a |  |  |  |
| tunel assay |  |  |  |
| neutrophil infiltration |  |  |  |
| drug delivery system |  |  |  |
| t lymphocyte |  |  |  |
| neutrophile extracellular trap |  |  |  |
| phorbol 13 acetate 12 myristate |  |  |  |
| fluorescent antibody technique |  |  |  |
| degranulation |  |  |  |
| microvascular thrombosis |  |  |  |
| protein blood level |  |  |  |
| protein phosphorylation |  |  |  |
| metabolic reprogramming |  |  |  |
| immunometabolism |  |  |  |
| homeostasis |  |  |  |
| glycolysis |  |  |  |
| immune system |  |  |  |
| cytotoxicity |  |  |  |
| interleukin 18 |  |  |  |
| molecular biology |  |  |  |
| cytokine release |  |  |  |
| lipopolysaccharide induced septic shock |  |  |  |
| mitogen activated protein kinase p38 |  |  |  |
| protein arginine deiminase type 2 |  |  |  |
| padi2 protein |  |  |  |
| thrombomodulin |  |  |  |
| interleukin 1beta converting enzyme |  |  |  |
| cryopyrin |  |  |  |
| hematoxylin |  |  |  |
| electron microscopy |  |  |  |
| antioxidant |  |  |  |
| von willebrand factor |  |  |  |
| anticoagulation |  |  |  |
| monocyte chemotactic protein 1 |  |  |  |
| microscopy |  |  |  |
| padgem protein |  |  |  |
| fluorescence microscopy |  |  |  |
| gram negative sepsis |  |  |  |
| immunofluorescence microscopy |  |  |  |
| algorithm |  |  |  |
| leukocyte function |  |  |  |
| klebsiella infections |  |  |  |
| intravital microscopy |  |  |  |
| extracellular matrix |  |  |  |
| confocal microscopy |  |  |  |
| kupffer cell |  |  |  |
| host resistance |  |  |  |
| length of stay |  |  |  |
| angiopoietin 2 |  |  |  |
| tumor necrosis factor alpha |  |  |  |
| drug efficacy |  |  |  |
| liposome |  |  |  |
| autophagy (cellular) |  |  |  |
| acute respiratory distress syndrome |  |  |  |
| fibrinogen |  |  |  |
| binding affinity |  |  |  |
| interleukin 12 |  |  |  |
| lipopolysaccharide induced sepsis |  |  |  |
| histone h4 |  |  |  |
| injury |  |  |  |
| drug targeting |  |  |  |
| heparin |  |  |  |
| cell damage |  |  |  |
| enzymology |  |  |  |
| microrna |  |  |  |
| anakinra |  |  |  |
| gasdermin |  |  |  |
| protein arginine deiminase |  |  |  |
| enzyme activation |  |  |  |
| wild type mouse |  |  |  |
| bacteria |  |  |  |
| bacterial infection |  |  |  |
| macromolecule |  |  |  |
| macromolecular substances |  |  |  |
| bacterial virulence |  |  |  |
| antithrombin |  |  |  |
| cardiomyopathy |  |  |  |
| cell function |  |  |  |
| bacteremia |  |  |  |
| biological model |  |  |  |
| negibacteria |  |  |  |
| bacterial count |  |  |  |
| autophagy |  |  |  |
| bacterial pneumonia |  |  |  |
| endothelium |  |  |  |
| autacoid |  |  |  |
| analogs and derivatives |  |  |  |
| chemokine receptor cxcr2 |  |  |  |
| cd11b antigen |  |  |  |
| cell-free dna |  |  |  |
| immunoglobulin |  |  |  |
| citrullinated histones |  |  |  |
| carboxy terminal sequence |  |  |  |
| case control study |  |  |  |
| blood analysis |  |  |  |
| cardiovascular disease |  |  |  |
| adherent cell |  |  |  |
| alanine transaminase |  |  |  |
| adolescent |  |  |  |
| biological markers |  |  |  |
| blood vessels |  |  |  |
| ligand |  |  |  |
| chemotaxis |  |  |  |
| cell activity |  |  |  |
| liver sinusoid |  |  |  |
| bacterial cell wall |  |  |  |
| immobilized cell |  |  |  |
| bacterial survival |  |  |  |
| epithelium |  |  |  |
| bacterium |  |  |  |
| bacterium pilus |  |  |  |
| blood vessel |  |  |  |
| bacterial colonization |  |  |  |
| bacterium adherence |  |  |  |
| bacterial infections |  |  |  |
| bacterial strain |  |  |  |
| bactericidal permeability increasing protein |  |  |  |
| immune evasion |  |  |  |
| cell adhesion |  |  |  |
| cathelicidin |  |  |  |
| lung capillary |  |  |  |
| ascorbic acid deficiency |  |  |  |
| toll like receptor 4 |  |  |  |

① = Consistency between singular and plural forms

② = Standardization of spelling variants

③ = Remove parenthetical abbreviations and retain the full names.

④ = Synonyms

⑤ = Consistency between abbreviations and full names

⑥ = Spelling correction

⑦ = Remove bracketed notes

⑧ = Standard interchangeable discipline names

⑨ = Standardization of hyphen usage

**Supplementary Table S3. Annual publication counts from Scopus, 2007-2012.**

| **Year** | **Publications** |
| --- | --- |
| 2007 | 1 |
| 2008 | 2 |
| 2010 | 1 |
| 2011 | 1 |
| 2012 | 10 |

**Supplementary Table S4. Overlap of the top 20 author keywords between WoSCC and Scopus.**

| **WoSCC TOP 20** | **Scopus TOP 20** |
| --- | --- |
| sepsis | sepsis |
| neutrophil extracellular traps (nets) | neutrophil extracellular traps |
| netosis | neutrophil |
| inflammation | netosis |
| neutrophil | inflammation |
| activation | acute lung injury |
| platelet | platelet |
| acute lung injury | disseminated intravascular coagulation |
| septic shock | septic shock |
| infection | pyroptosis |
| mortality | biomarker |
| coagulation | acute respiratory distress syndrome |
| receptor | cell-free dna |
| innate immunity | coagulopathy |
| expression | immunothrombosis |
| apoptosis | infection |
| extracellular traps | innate immunity |
| immunity | apoptosis |
| mechanism | coagulation |
| release | reactive oxygen species |
